# “Double-Use” Strategy for Improving the Photoelectrochemical Performance of BiVO_4_ Photoanodes Using a Cobalt-Functionalized Polyoxotungstate

**DOI:** 10.1021/acsami.4c21125

**Published:** 2024-12-30

**Authors:** Fan Feng, Dariusz Mitoraj, Ekemena Oseghe, Carsten Streb, Radim Beranek

**Affiliations:** † Department of Chemistry, Johannes Gutenberg University Mainz, Duesbergweg 10-14, Mainz 55128, Germany; ‡ Institute of Electrochemistry, Ulm University, Albert-Einstein-Allee 47, Ulm 89081, Germany

**Keywords:** BiVO_4_, polyoxometalate, oxygen evolution, photoelectrode, photoelectrocatalysis

## Abstract

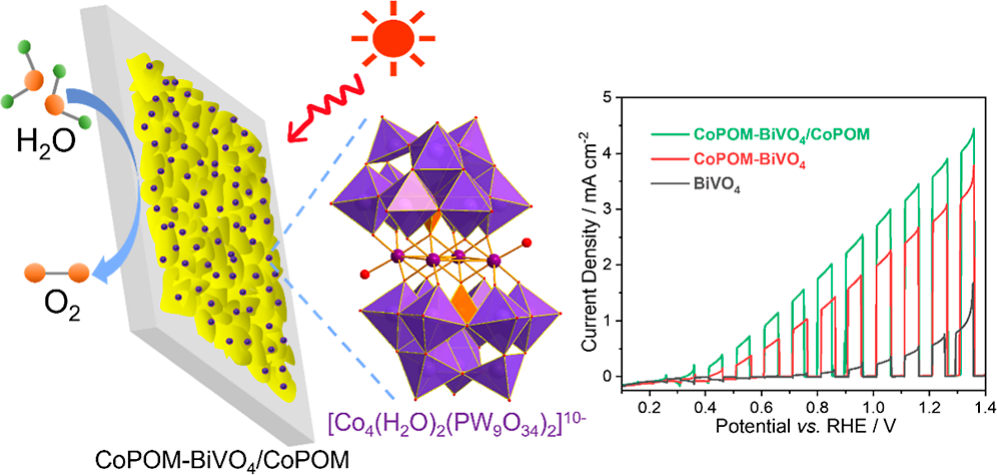

Doping and surface-modification are well-established strategies for the performance enhancement of bismuth vanadate (BiVO_4_) photoanodes in photoelectrochemical (PEC) water splitting devices. Herein, a “double-use” strategy for the development of high-performance BiVO_4_ photoanodes for solar water splitting is reported, where a molecular cobalt–phosphotungstate (CoPOM = Na_10_[Co_4_(H_2_O)_2_(PW_9_O_34_)_2_]) is used both as a bulk doping agent as well as a surface-deposited water oxidation cocatalyst. The use of CoPOM for bulk doping of BiVO_4_ is shown to enhance the electrical conductivity and improve the charge separation efficiency, resulting in the enhancement of the maximum applied-bias photoconversion efficiency (ABPE) by a factor of ∼18 to 0.54% at 0.87 V vs. RHE, as compared to pristine BiVO_4_ (0.03% at 1.04 V vs. RHE). The ratio of W/Co on the surface of the photoanode is related to the activity and stability. In addition, modification of CoPOM-doped BiVO_4_ with CoPOM as a surface cocatalyst enhances the hole extraction and improves the water oxidation kinetics, resulting in the overall enhancement of the ABPE to 0.79% (at 0.82 V vs. RHE), i.e., by a factor of ∼26 with respect to pristine BiVO_4_. This study establishes the “double-use” strategy involving CoPOMs as an effective, straightforward, and easily scalable approach for the development of high-quality photoanodes for solar water splitting and highlights the future potential of utilizing well-designed polyoxometalates as precursors for the synthesis of energy materials.

## 1 Introduction

Photoelectrochemical (PEC) water splitting using semiconductor photoelectrodes is regarded as a promising and sustainable strategy to address the future supply of energy in a sustainable way.^1,2^ However, the search for robust and efficient photoanode materials for water oxidation remains one of the biggest challenges for practical applications.^3,4^ Bismuth vanadate (BiVO_4_) is one of the most promising photoanode materials as it combines a suitable bandgap (ca. 2.4–2.6 eV) and good stability under typical operating conditions in aqueous solutions.^5,6^ When compared with other metal oxides, such as α-Fe_2_O_3_, BiVO_4_ exhibits charge carrier lifetimes that are 4 orders of magnitude higher and hole diffusion length 1 order of magnitude longer.^7−9^ Nevertheless, pristine BiVO_4_ still suffers from poor bulk electronic conductivity, ineffective hole extraction, and a slow rate of water oxidation.^7,10^ As a result, the reported photocurrent densities at nonmodified BiVO_4_ are typically much lower than the maximum theoretical value of 6.4–8.9 mA cm^–2^ under AM 1.5G illumination, corresponding to maximum solar-to-H_2_ conversion efficiencies of 8–11%.^11,12^ Thus, designing straightforward, scalable strategies to overcome these drawbacks of BiVO_4_ materials represents an important research agenda, whereby the best-performing BiVO_4_-based photoanodes reported so far typically employed doping by molybdenum or tungsten combined with surface functionalization by Ni/Fe-(oxy)hydroxides as cocatalysts.^13−16^

One recent approach to modify BiVO_4_ photoelectrodes is the incorporation of polyoxometalates (POMs) as single-source molecular precursors. POMs are molecular metal oxide clusters formed by self-assembly in solution.^17−19^ Of specific interest in this context is the cobalt-functionalized polyoxometalate [Co_4_(H_2_O)_2_(PW_9_O_34_)_2_]^10–^ (CoPOM), where a {Co_4_O_4_} core is stabilized by two oxidatively resistant polyoxotungstate ligands.^20−22^ The compound has been reported as an active water oxidation catalyst (WOC) as well as precursor to highly reactive cobalt oxyhydroxides, so that operation under homogeneous or heterogenized conditions is possible.^19,23^ This in turn has attracted major interest for surface-deposition as cocatalyst on BiVO_4_ photoanodes. For example, Ryu et al. successfully designed a photoanode by depositing a thin film of cationic polyelectrolytes and anionic CoPOM-WOCs on the surface of various photoelectrode materials (e.g., Fe_2_O_3_, BiVO_4_, and TiO_2_) for improving the PEC performance as well as stability.^24^ Fan et al. reported CoPOM as a novel molecular cocatalyst deposited on N-doped carbon (N/C) to boost the charge separation and injection of BiVO_4_ photoanodes for PEC water oxidation.^25^ Some of us have recently reported direct linking of CoPOMs to the surface of Mo-doped BiVO_4_ photoanodes. The system showed significantly enhanced PEC performance, featuring lower photocurrent onset potentials and higher charge transfer efficiency.^26^ These initial studies suggest that CoPOM is a promising cocatalyst for PEC water oxidation at BiVO_4_ photoanodes as it improves the photogenerated hole extraction and enhances the oxygen evolution reaction kinetics. This inspired the present study to explore how a “double-use” of bulk and surface deposition of CoPOM can be used to optimize the performance.

Notably, transition metal doping is an established strategy to improve the conductivity of BiVO_4_ and promote PEC performance. For example, He et al. developed BiVO_4_ nanoflake array films codoped with Mo and W to increase the conductivity of BiVO_4_ and to slightly to enhance the water oxidation kinetics.^27^ Fang et al. reported that cobalt ion-doped BiVO_4_ photoanodes exhibit an improved PEC performance, rationalized by the fact that a part of Co ions are doped into the BiVO_4_ lattice to increase its electronic conductivity, whereby excess Co ions form a Co_3_O_4_ cocatalyst on the BiVO_4_ surface.^28^ Shi et al. reported that W-doped BiVO_4_ shows a higher donor density and a higher concentration of surface states that can act as reaction sites, leading thus to improvements of both conductivity and surface catalytic activity.^29^ To date, using POMs as a doping agent has been reported, for example, in the fields of gas sensing application,^30,31^ dye-sensitized^32^ or perovskite^33,34^ solar cells, thiol detection,^35^ and photocatalysis.^36^ Due to the abundancy of transition metals involved in typical POMs, they also hold potential as doping agents in PEC water splitting systems. In view of enhancing the electron extraction and transport from BiVO_4_ to the underlying FTO conductive glass substrate, Xi et al. fabricated composite photoanodes consisting of BiVO_4_ and selected POMs, such as [H_3_PW_12_O_40_] (PW_12_) and K_6_[CoW_12_O_40_] (CoW_12_).^37^ However, CoPOMs have rarely been used as doping agent to manipulate the electronic structure of BiVO_4_ film photoanodes in a PEC water splitting system, and—to the best of our knowledge—CoPOM has not been used both as a dopant as well as a cocatalyst for BiVO_4_ photoanodes.

Herein, we report a “double-use” strategy to modify BiVO_4_ photoanodes using CoPOM simultaneously as both a doping agent and a cocatalyst in BiVO_4_ photoanodes. We show that the combination of both modification routes results in significantly increased photocurrent densities compared with pristine (nonmodified) BiVO_4_ and discuss in detail the mechanistic aspects of the improved activity and stability. These results establish the “double-use” strategy involving CoPOMs as a facile and scalable approach for the development of photoanodes for PEC water splitting.

## 2 Experimental Section

### 2.1 Materials

Fluorine-doped tin oxide (FTO) Pilkington TEC glass was purchased from XOP (XOP Glass, Castellón, Spain). Ethylene glycol (C_2_H_6_O_2_, ≥99%) and boric acid (H_3_BO_3_, 99.5%) were provided by Carl Roth Gmbh & Co. KG. Sodium hydroxide (NaOH, 98.7%) was obtained from Fisher Scientific. Cobalt (II) nitrate hexahydrate (Co(NO_3_)_2_·6H_2_O, 98%), bismuth (III) nitrate pentahydrate (Bi(NO_3_)_3_·5H_2_O, ≥98.0%), triblock copolymer F-108, sodium sulfite (Na_2_SO_3_, 98%), and sodium phosphate (Na_2_HPO_4_, 99 + %) were supplied by Sigma-Aldrich. Sodium tungstate dihydrate (NaWO_4_·2H_2_O) was provided by Merck. Vanadyl (IV) acetylacetonate (C_10_H_14_O_5_V, 99%) was obtained from Acros Organics. Hydrochloric acid (HCl_aq_, 37%), glacial acetic acid (CH_3_COOH, 100%), and sodium chloride (NaCl, 99.7%) were purchased from VWR.

### 2.2 Synthesis of CoPOM

The CoPOM complex was synthesized using a slightly modified literature route.^19^ Briefly, NaWO_4_·2H_2_O (35.62 g, 0.108 mol), Na_2_HPO_4_·7H_2_O (1.70 g, 0.012 mol), and Co(NO_3_)_2_·6H_2_O (6.98 g, 0.024 mol) were dissolved in 100 mL of deionized water in a 200 mL round-bottom flask. After the pH was adjusted to 7 using 9 M aqueous HCl solution under magnetic stirring, the purple suspension was stirred and refluxed at 105 °C bath temperature for 2 h. After reflux, the solution was saturated with 36 g of NaCl and allowed to cool to room temperature. The resulting purple crystals were collected, quickly washed with approximately 30 mL of water, and recrystallized from hot water. The purity of CoPOM was confirmed by Fourier transform infrared spectroscopy (FT-IR).

### 2.3 Preparation of CoPOM-Doped-BiVO_4_ (Labeled **Composite 1**)

In a typical synthetic procedure, a solution of 0.15 M Bi(NO_3_)_3_·5H_2_O (0.2910 g, 0.6 mmol) was prepared in a solvent mixture of 1.5 mL of ethylene glycol, 2 mL of glacial acetic acid, 100 μL of 5 mM CoPOM aqueous solution (0.0027 g, 5 × 10^–7^ mol), and 400 μL of deionized water for **Composite 1**. (For the pure BiVO_4_ reference, a similar synthetic mixture solvent was used containing 1.5 mL of ethylene glycol, 2 mL of glacial acetic acid, and 500 μL of deionized water. Other processes are the same as those for **Composite 1** samples.) After the mixture was stirred for 15 min, VO(acac)_2_ (0.3182 g, 1.2 mmol) was added into the above solution, and the resulting solution was stirred at room temperature for 1 h. Then, 0.35 g triblock copolymer (F-108) as structure-directing agent was added in this solution, and the resulting solution was stirred at room temperature for 2 h. The resulting precursor solution was deposited on cleaned FTO glasses (2 × 2 cm) by spin-coating.^38^ Spin-coating was performed for the ink precursor solution (75 μL per run) at 50 rps for 30 s, followed by baking at 250 °C for 5 min using a hot plate and then cooling down to room temperature naturally; the above coating and baking process was repeated five times. Finally, samples were annealed in a muffle oven with a heating rate of 3 °C/min and kept at 450 °C for 1 h, cooling down to room temperature naturally. After sintering, the sample was immersed in 1 M aqueous NaOH solution for 40 min to remove the excess V_2_O_5_, rinsed with deionized water, and dried with air flow. Different CoPOM-doping amount thin films were synthesized via the same procedure, except added amounts (50 μL, 150 μL, and 200 μL) of CoPOM aqueous solution (5 mM) in the solvent mixture were used instead of 100 μL. Deionized water and CoPOM aqueous solution in the solvent mixture make up a total of 500 μL, for example, 50 μL of CoPOM aqueous solution and 450 μL of deionized water in the mixture solvent for the CoPOM-BiVO_4_ (50 μL) sample.

### 2.4 Preparation of CoPOM-Doped-BiVO_4_/CoPOM (Labeled **Composite 2**)

The **Composite 1** electrode was immersed in 50 mL 5 mM aqueous CoPOM solution (1.35 g, 2.5 × 10^–4^ mol) for 30 min, rinsed with distilled water, and dried in air flow. Different immersing time thin films were synthesized via the same procedure, except different immersing times (10 min and 60 min) were used instead of 30 min.

### 2.5 Preparation of Co/W/P-BiVO_4_ (Labeled **Composite 3**)

As a reference sample, a BiVO_4_ photoanode containing the CoPOM components Co(II), W(VI), and P(V) was prepared as follows. A solvent mixture was prepared containing 1.5 mL ethylene glycol, 2 mL glacial acetic acid, 100 μL 20 mM Co(NO_3_)_2_·6H_2_O (0.58 mg, 2 × 10^–6^ mol) aqueous solution, 100 μL 90 mM NaWO_4_·2H_2_O (2.97 mg, 9 × 10^–6^ mol) aqueous solution, 100 μL 10 mM Na_2_HPO_4_·7H_2_O (0.14 mg, 1 × 10^–6^ mol) aqueous solution, and 200 μL deionized water at room temperature. Other processes are the same as for **Composite 1** samples.

### 2.6 Preparation of Co/W-BiVO_4_

A solvent mixture was prepared containing 1.5 mL ethylene glycol, 2 mL glacial acetic acid, 100 μL 20 mM Co(NO_3_)_2_·6H_2_O (0.58 mg, 2 × 10^–6^ mol) aqueous solution, 100 μL 90 mM NaWO_4_·2H_2_O (2.97 mg, 9 × 10^–6^ mol) aqueous solution, and 300 μL deionized water at room temperature. Other processes are the same as for **Composite 1** samples.

### 2.7 Preparation of Co/P-BiVO_4_

A solvent mixture was prepared containing 1.5 mL ethylene glycol, 2 mL glacial acetic acid, 100 μL 20 mM Co(NO_3_)_2_ 6H_2_O (0.58 mg, 2 × 10^–6^ mol) aqueous solution, 100 μL 10 mM Na_2_HPO_4_ 7H_2_O (0.14 mg, 1 × 10^–6^ mol) aqueous solution, and 300 μL deionized water at room temperature. Other processes are the same as for **Composite 1** samples.

### 2.8 Preparation of W/P-BiVO_4_

A solvent mixture was prepared containing 1.5 mL of ethylene glycol, 2 mL of glacial acetic acid, 100 μL of 90 mM NaWO_4_·2H_2_O (2.97 mg, 9 × 10^–6^ mol) aqueous solution, 100 μL of10 mM Na_2_HPO_4_·7H_2_O (0.14 mg, 1 × 10^–6^ mol) aqueous solution, and 300 μL of deionized water at room temperature. Other processes are the same as for **Composite 1** samples.

### 2.9 Preparation of Co-BiVO_4_

A solvent mixture was prepared containing 1.5 mL of ethylene glycol, 2 mL of glacial acetic acid, 100 μL of 20 mM Co(NO_3_)_2_ 6H_2_O (0.58 mg, 2 × 10^–6^ mol) aqueous solution, and 400 μL of deionized water at room temperature. Other processes are the same as for **Composite 1** samples.

### 2.10 Preparation of W-BiVO_4_

A solvent mixture was prepared containing 1.5 mL of ethylene glycol, 2 mL of glacial acetic acid, 100 μL of 90 mM NaWO_4_·2H_2_O (2.97 mg, 9 × 10^–6^ mol) aqueous solution, and 400 μL of deionized water at room temperature. Other processes are the same as for **Composite 1** samples.

### 2.11 Preparation of P-BiVO_4_

A solvent mixture was prepared containing 1.5 mL of ethylene glycol, 2 mL of glacial acetic acid, 100 μL of 10 mM Na_2_HPO_4_·7H_2_O (0.14 mg, 1 × 10^–6^ mol) aqueous solution, and 400 μL of deionized water at room temperature. Other processes are the same as for **Composite 1** samples.

### 2.12 Characterization

The UV–vis absorption spectra were determined with a UV–vis spectrophotometer (UV-2600, Shimadzu, Japan) equipped with an integrating sphere. The absorbance (Abs) was calculated as follows
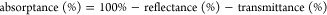
1

The baselines were recorded using an FTO glass and a BaSO_4_ plate as references for transmittance and reflectance, respectively. Solid-state photoluminescence (PL) spectra were recorded under an excitation wavelength of 400 nm using a Shimadzu RF-6000 spectrometer. Scanning electron microscopy (SEM) was performed using a LEO Gemini 1530 scanning electron microscope operating at an acceleration voltage of 3 kV. The cross-section images of SEM were taken 75° pretilt for all photoanodes. Transmission electron microscopy (TEM) and energy-dispersive X-ray spectroscopy (EDX) elemental mapping were performed with FEI. FT-IR was performed on a Bruker Tensor 27 equipped with a PIKE Miracle Diamond ATR unit. X-ray photoelectron spectroscopy (XPS) measurements were performed with monochromatized Al Kα radiation (250 W, 15 kV) by using a PHI 5800 ESCA system. The binding energies were calibrated based on the C 1s peak of adventitious carbon (284.8 eV). X-ray diffraction (XRD) patterns were recorded on a Rigaku XRD-6000 diffractometer under the following conditions: 40 kV, 40 mA, and Cu Kα radiation (λ = 0.154 nm).

### 2.13 Photoelectrochemical Measurements

The PEC measurements were conducted using a CHI 760E electrochemical workstation or an SP-300 BioLogic potentiostat in a typical 3-electrode system with a Pt wire as the counter electrode, an Ag/AgCl (1.0 M KCl, 0.222 V vs. SHE) as the reference electrode, and film photoanodes as the working electrodes with a geometric irradiation area of 0.5 cm^2^. A 150 W Xe lamp (L.O.T.-Oriel) equipped with a KG-3 (LOT-Quantum Design) heat-absorbing filter and an AM 1.5G filter was employed as the light source (light power intensity 100 mW cm^–2^). The electrolyte was 0.5 M aqueous sodium borate, pH = 9.0. All electrodes were illuminated from the backside (through the FTO glass) unless noted otherwise.

All electrode potentials reported were converted to the RHE following the equation^39^
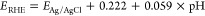
2where *E*_RHE_ is the pH-independent potential referenced against RHE, and *E*_Ag/AgCl_ is the experimentally determined electrode potential referenced against an Ag/AgCl reference electrode.

The charge separation efficiency (η_sep_) and the hole transfer efficiency (η_tr_) were calculated using the approach reported by Dotan et al.^40^ and Hamann et al.^41^ The hole transfer efficiency (η_tr_) was calculated using the equation
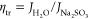
3where  is the photocurrent measured in the absence of a hole scavenger, and  is the photocurrent in the presence of a hole scavenger (Na_2_SO_3_, 0.1 M).

The charge separation efficiency (η_sep_) is calculated using the equation
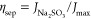
4where *J*_max_ is the maximal photocurrent density obtained by integrating the absorption spectrum of the photoanode (Figure S8, Supporting Information) between 300 and 560 nm over the reference AM 1.5G photon flux spectra (https://www.nrel.gov/grid/solar-resource/spectra-am1.5.html).

The incident photon-to-current conversion efficiency (IPCE) was recorded using a photoelectric spectrometer (Instytut Fotonowy Sp. z o.o.) with a 150 W xenon light source equipped with a monochromator, according to the equation^38^
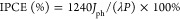
5where *J*_ph_ is the photocurrent density under monochromatic light, *P* is the monochromatic light power density, and *λ* is the irradiation wavelength.

The applied bias photoconversion efficiency (ABPE) was calculated using the equation^42^
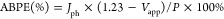
6where *J*_ph_ is the photocurrent density, *V*_app_ is the applied bias (V vs. RHE), and *P* is the incident light intensity (100 mW cm^–2^).

The transient decay time can be calculated from a logarithmic plot of parameter *D*, given by the equation^43^
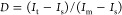
7where *I*_m_ is the photocurrent spike, *I*_t_ is the photocurrent at time t, and *I*_s_ is the steady-state photocurrent. *I*_s_ is achieved as the recombination and charge generation reach equilibrium. The transient decay time is defined as the time at which ln *D* = −1.

Oxygen evolution was detected using a FireSting optical fiber oxygen meter (PyroScience, GmbH) in a homemade airtight two-compartment cell. The oxygen collection efficiency of approximately 42.6% ± 1.6% was determined electrochemically using a Pt working electrode in a 3-electrode system and assuming a standard faradaic efficiency (FE, based only on dissolved O_2_) of 100.0% ± 3.8%. The applied potential was 0.80 V vs RHE, and the electrolyte was bubbled with argon before the measurement.

## 3 Results and Discussion

**Scheme 1:**
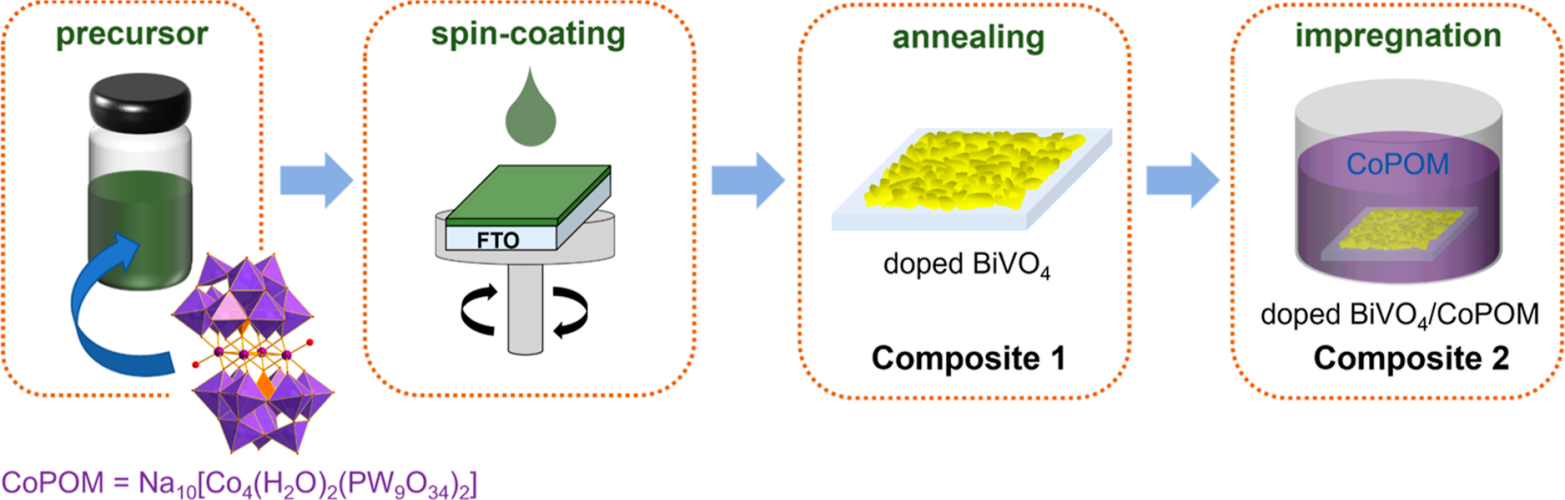
“Double-Use” Strategy Utilizing CoPOM for Improved BiVO_4_ Photoanodes: CoPOM Is Added to a Precursor Solution that Is Spin-Coated onto FTO-Glass Substrate and Annealed to Yield Doped BiVO_4_ (= **Composite 1**); Further Impregnation by CoPOM Yields Doped BiVO_4_ Surface-Modified with CoPOM (= **Composite 2**)

After sample preparation as described in Scheme 1 and the Experimental Section, the phase purity and crystallinity of the BiVO_4_ photoanodes were analyzed by powder X-ray diffraction (pXRD) (Figure S1, Supporting Information). All diffraction peaks can be indexed to monoclinic scheelite BiVO_4_ (JCPDS card number 14-0688)^38^ and to the FTO support. No crystalline impurities (such as V_2_O_5_) were detected. Moreover, no diffraction peaks associated with the CoPOM compound were observed. These observations highlight that the reported synthetic approach leads to thin-film BiVO_4_ photoanodes, whereby the doping and surface-modification with CoPOM do not significantly alter the crystal lattice of the resulting system.

XPS was used to assess the chemical environment and oxidation states of the relevant elements. XPS survey analysis indicates the presence of Bi, V, O, Co, W, and Sn signals on the surface of **Composite 2** photoanodes (Figure S2a, Supporting Information). The Sn signal is due to the use of FTO as a conductive transparent support.^44,45^ The deconvoluted Bi 4f and V 2p spectra show the characteristic peaks for Bi^3+^ (at 164.9 and 159.6 eV for Bi 4f_5/2_ and Bi 4f_7/2_, respectively) and V^5+^ (at 522.7 eV for V 2p_1/2_ and 515.2 eV for V 2p_3/2_) expected for BiVO_4_ (Figure S2b,c, Supporting Information).^46,47^ In the O 1s spectrum, the two characteristic peaks located at 528.3 and 529.5 eV (Figure S2d, Supporting Information) are assigned to lattice oxygen in bismuth vanadate and surface hydroxyl oxygen, respectively.^48,49^ Compared with pristine BiVO_4_, the Bi 4f, V 2p, and O 1s peaks of **Composite 1** are shifted to higher binding energies by 0.1, 0.4, and 0.4 eV, respectively (Figure S2b–d, Supporting Information). This can be explained by interactions of dopant elements (Co^3+^, W^6+^, see below XPS analysis) from CoPOM with Bi, V, and O atoms. The slight shifts are expected due to the higher charge-densities of the dopants (W^6+^ > V^5+^, Co^3+^ > Bi^3+^).^50−52^ These data support the successful incorporation of the CoPOM components (W^6+^, Co^3+^) into the BiVO_4_ lattice. Note that similar shifts in binding energies are observed for the **Composite 3** reference photoanode (Figure S2b–d, Supporting Information).

**Figure 1:**
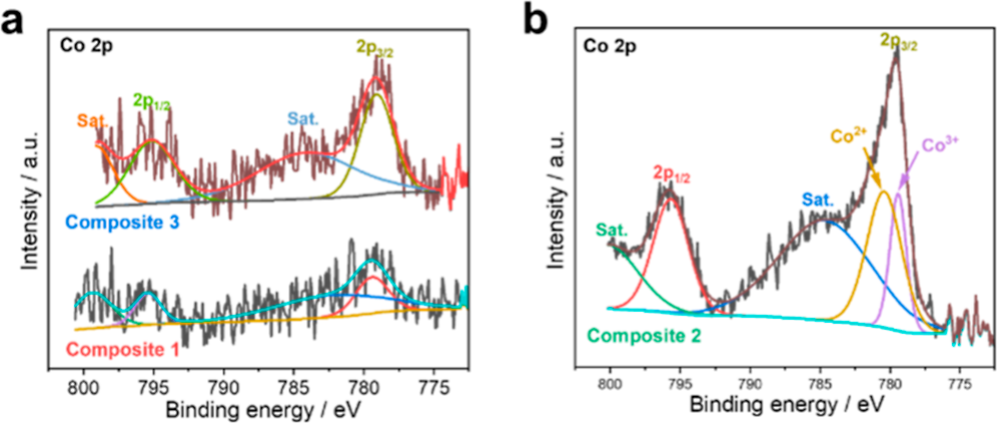
Co 2p XP spectra for (a) **Composite 1** and **Composite 3** and (b) **Composite 2** photoanodes.

To discriminate between the chemical states of elements originating from CoPOM and present either as a dopant or a cocatalyst, XPS analysis has been carried out, and the Co 2p and W 4f XP spectra are shown in Figures 1 and S3 (Supporting Information). As a reference, XPS analysis of the pristine CoPOM powder shows, as expected, the presence of Co^2+^ species at the binding energies of 797.0 and 781.1 eV for the Co 2p_1/2_ and Co 2p_3/2_ spin–orbit coupling peaks, respectively (Figure S4a, Supporting Information).^26^ In contrast, the low-intensity Co 2p XPS data of the doped **Composite 1** sample (Figure 1a) show the Co 2p_1/2_ and Co 2p_3/2_ peaks located at 795.2 and 779.1 eV, which indicates the presence of Co^3+^.^39,53^ This suggests that Co^2+^ from the CoPOM dopant is oxidized to Co^3+^ during the photoanode annealing process. After additional surface modification with CoPOM, the resulting **Composite 2** photoanode exhibits signals characteristic of both Co^2+^ and Co^3+^ (Figure 1b). Taken together, these data indicate that while the Co dopant within the BiVO_4_ bulk is present as Co^3+^, the surface-deposited CoPOM retains its original Co^2+^ oxidation state, which is also in line with our previous study on CoPOM as cocatalyst.^26^ In addition, the oxidation state of tungsten in all samples determined from the high-resolution XP spectra of W 4f was W^6+^ (Figure S3, Supporting Information), as expected for CoPOM derivatives.^25,54^

**Figure 2:**
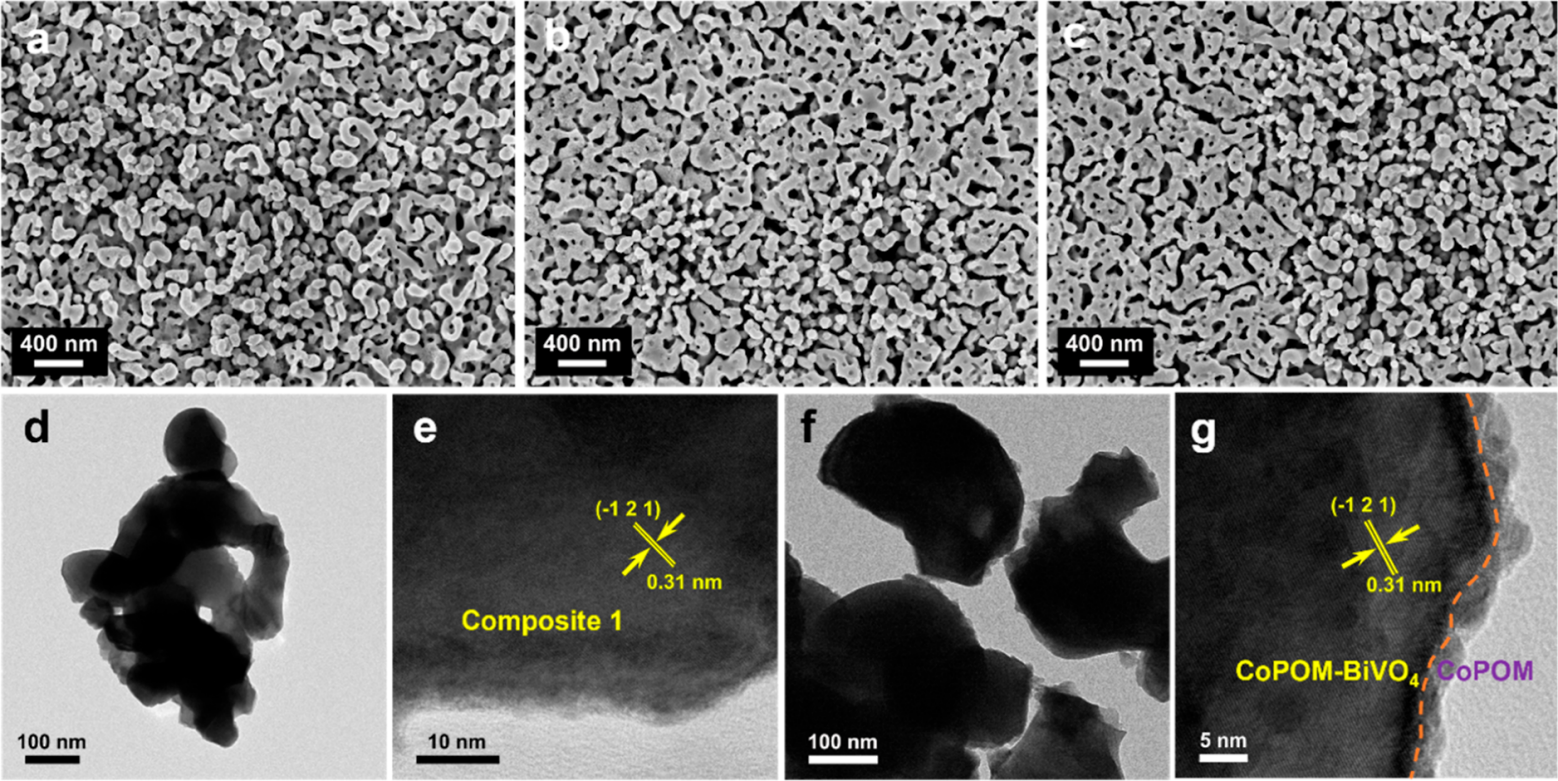
SEM images of (a) BiVO_4_, (b) **Composite 1**, and (c) **Composite 2**. TEM and high-resolution TEM images for (d,e) **Composite 1** and (f,g) **Composite 2**.

The morphology and film thickness of as-prepared BiVO_4_ and **Composite 1** and **Composite 2** photoanodes were investigated by field emission scanning electron microscopy (FESEM). As shown in Figure 2a–c, all of the films are composed of worm-like nanoparticles forming a three-dimensional mesoporous structure. After the CoPOM doping and surface modification with CoPOM, the worm-like particle shape does not change significantly, as shown in Figure 2b,c, retaining the typical porous structure. However, after the CoPOM doping of BiVO_4_, the average length of worm-like particles increases from 160 to 277 nm (see Figure S5d,e, Supporting Information) and the particles become more interconnected. The cross-sectional views (Figure S6, Supporting Information) show that the BiVO_4_ layers have a comparable thickness of 520 ± 40 nm in all photoanodes. High-resolution transmission electron microscopy (HR-TEM) of **Composite 1** and **Composite 2** shows the presence of crystalline regions with a lattice d-spacing of 0.31 nm, which can be assigned to the (−121) plane of BiVO_4_ (JCPDS 14-0688) (Figure 2e,g). As compared to **Composite 1** photoanodes (Figure 2e), additional surface modification with CoPOM leads to the presence of homogeneously distributed nanospheres with a size of ∼5 nm, as indicated by the orange dotted lines in Figure 2g. This suggests the formation of a CoPOM-derived surface layer on the **Composite 1** photoanodes, in line with our previous results.^[^^21^^]^ To investigate the distribution of CoPOM, EDX elemental mapping was carried out. This analysis (Figure S7, Supporting Information) indicates the presence of Co and W elements, which is in line with the homogeneous distribution of CoPOM within the BiVO_4_ bulk and on the BiVO_4_ surface since the structure of the **Composite 1** material is porous and the CoPOM was deposited via impregnation.

The UV–vis electronic absorption spectra of all four samples displayed in Figure S8, Supporting Information, indicate that all samples exhibit nearly identical electronic absorption properties with an optical absorption edge at ∼530 nm. Therefore, we conclude that there is a negligible influence of either CoPOM-doping or Co/W/P-doping or the deposition of the CoPOM cocatalyst on the fundamental optical properties of our photoanodes, and all differences in photoelectrocatalytic behavior should be rather ascribed to changes in the electronic conductivity and surface catalytic properties.

**Figure 3:**
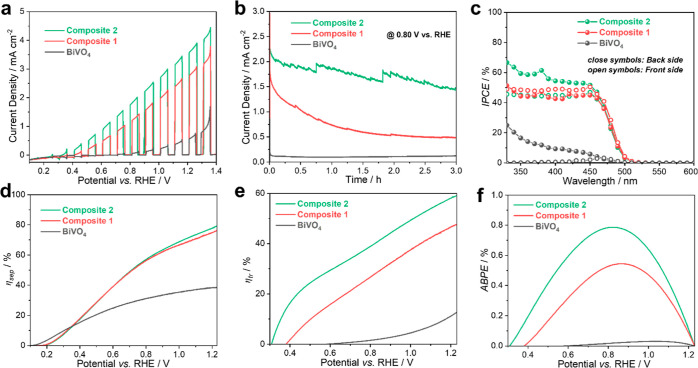
(a) Photocurrents recorded under AM 1.5G illumination in borate electrolyte at anodic sweep of 10 mV s^–1^; (b) chronoamperometry curves under AM 1.5G illumination at 0.80 V vs. RHE; (c) IPCE spectra under intermittent monochromatic irradiation; (d) charge separation efficiency (η_sep_), (e) hole transfer efficiency (η_tr_), (f) ABPE plots for BiVO_4_ and **Composite 1** and **Composite 2** photoanodes.

To evaluate the performance of our photoanodes in water photooxidation, PEC measurements were conducted in a three-electrode setup under AM 1.5G (1 sun) illumination with a 0.5 M aqueous sodium borate solution (pH 9.0) as electrolyte. The photoanodes were optimized for the best PEC performance with respect to the amount of CoPOM used either as a bulk dopant and/or surface cocatalyst, as shown in Figure S9a,b, Supporting Information. As shown in Figure 3a, the pristine BiVO_4_ photoanodes exhibit a relatively low photocurrent density (0.62 mA cm^–2^ at 1.23 V vs. RHE), which is mainly attributed to the poor electronic conductivity and sluggish oxygen evolution kinetics at the photoanode/electrolyte interfaces.^7,10^ After CoPOM-doping, for **Composite 1**, a significantly improved photocurrent density (5-fold increase compared with pristine BiVO_4_) is observed, reaching 2.86 mA cm^–2^ at 1.23 V vs. RHE. When the surface of the sample is further loaded with CoPOM as cocatalyst, the photocurrent density is further increased by a factor of ∼1.3 to 3.67 mA cm^–2^ at 1.23 V vs. RHE. Importantly, we observe a cathodic shift of the photocurrent onset potential by ∼0.3 V for **Composite 2** and ∼0.2 V for **Composite 1** compared with the nonmodified BiVO_4_ photoanode (Figure 3a, see also Figure S10, Supporting Information). In other words, both CoPOM-doping and surface modification by CoPOM result in a significant increase of the photovoltage available for driving the water splitting reaction.^20^ The reasons for this performance enhancement are discussed below. The dark current densities are similar for **Composite 1**, **Composite 2**, **Composite 3**, and BiVO_4_ (Figure S10, Supporting Information) below the equilibrium potential of water oxidation (1.23 V vs. RHE), and the beneficial effects of CoPOM on electrocatalytic water oxidation reaction activity in the dark become apparent at bias potentials of >1.40 V vs. RHE.^55^

First, in order to gain insight into the role of the individual doping elements (Co, W, and P) present in CoPOM, we prepared also doped BiVO_4_ samples where NaWO_4_·2H_2_O, Na_2_HPO_4_·7H_2_O, and Co(NO_3_)_2_·6H_2_O were used as sources of the respective elements, and identical molar amounts were incorporated as in the case of **Composite 1** photoanodes (see Figures 3a and S9c, Supporting Information). Notably, Co-doping of BiVO_4_ exerts a positive influence on the photocurrent density also at lower electrode potentials (leading to the significant cathodic shift of the onset potential), while W-doping of BiVO_4_ enhances photocurrents significantly only at higher bias potentials (>0.7 V vs. RHE). Interestingly, there is a clear beneficial synergistic effect when both Co- and W-doping of BiVO_4_ are used. Furthermore, the photocurrent at the **Composite 1** photoanode is slightly higher than that of the **Composite 3** photoanode in the full potential range. The possible reason is that the ratio of W/Co on the surface of BiVO_4_ detected by XPS is different (2.29 for the **Composite 1** photoanode and 1.08 for the **Composite 3** photoanode), as shown in Table S1 (Supporting Information). We hypothesize that this difference in the W/Co ratio on the surface of the **Composite 1** and **Composite 3** photoanodes might be due to the different forms of the doping substances. For example, the structure of CoPOM is expected to be partially disintegrated but will still retain its core structure in the acid precursor solution, while the doping solution used for preparation of the reference **Composite 3** photoanode is fully homogeneous. Apparently, such small differences in the precursor chemistry can influence the W/Co ratio on the surface and impact the photocurrent generation.

Next, the photocurrent generation was tested at a constant potential of 0.80 V vs. RHE under prolonged (3 h) AM 1.5G (1 sun) illumination. As shown in Figure 3b, CoPOM-doped BiVO_4_ showed a relatively fast and continuous decrease of photocurrent. To explore the reasons for the instability of **Composite 1** under PEC operation, we analyzed in detail the elemental composition of the surface for CoPOM-doped BiVO_4_ before and after the PEC experiments, as shown in Figure S11 and Table S2, Supporting Information. Interestingly, the XPS analysis revealed that the signal related to the presence of Co is significantly stronger after PEC operation (Figure S11a, Supporting Information), while the signal of W practically did not change at all after PEC operation (Figure S11c, Supporting Information). Notably, the ratio of W/Co for the **Composite 1** photoanode significantly decreased after the PEC operation, from 2.29 for before PEC operation to 0.59 for after PEC operation (Table S2, Supporting Information). In other words, we see a similar trend as discussed above for the case of the **Composite 1** photoanode (higher ratio) and the **Composite 3** photoanode (lower ratio). We conclude that the PEC performance is related to the ratio of W/Co on the surface of photoanode, whereby a higher ratio of W/Co will result in better PEC performance. The underlying reasons are still under study. Previous reports indicated that W^6+^ sites can act as structure stabilizers to the OER active Co sites.^56^ Therefore, a possible reason for this trend might be that at the lower ratios of W/Co, the low amount of W cannot prevent the pronounced leaching of Co from the BiVO_4_ surface into the borate buffer electrolyte, leading eventually to the formation of cobalt borate (CoBi),^28^ which is known to exhibit operational instability in BiVO_4_/CoBi systems.^57^

Notably, employing CoPOM as a surface cocatalyst to modify the **Composite 1** photoanode turned out be highly beneficial as higher and much more stable photocurrents were observed (the green line in Figure 3b). The small jumps in the photocurrent were most probably due to the bubble release from the testing area, as observed visually during the PEC operation. We used XPS to analyze the elemental composition of the surface for **Composite 2** before and after the PEC experiments, as shown in Figure S11 and Table S3, Supporting Information. In contrast to **Composite 1**, the signals related to the presence of Co and W were significantly weaker after the PEC operation (Figure S11b,d, Supporting Information) with a slightly decreased ratio of W/Co for the **Composite 2** photoanode than before the PEC operation (Table S3, Supporting Information). In particular, the changes in signal intensities and ratio of W/Co indicate that a partial dissolution of the CoPOM cocatalyst and/or its conversion to cobalt and tungsten oxides can occur during the PEC operation, which is consistent with the findings discussed in detail in our previous study.^26^ As shown in Figure S12a of the Supporting Information, the **Composite 3** shows a slightly lower photocurrent density during a 3 h stability measurement compared with **Composite 1**, which is also consistent with the lower W/Co ratio on the surface of **Composite 3** photoanodes (Table S1, Supporting Information).

Furthermore, the transient photocurrent measurements of pristine BiVO_4_ and **Composite 1** and **Composite 2** photoanodes were investigated to assess the charge recombination dynamics at +0.80 V vs. RHE under chopped AM 1.5G (1 sun) illumination (Figure S12b, Supporting Information). The strong spike-like behavior of the photocurrent transients at pristine BiVO_4_, a typical fingerprint of intense surface recombination due to slowly reacting holes accumulating in the surface states,^58^ is clearly diminished to a large extent at the **Composite 1** and **Composite 2** samples, indicating reduced charge carrier recombination for these samples. In more quantitative terms, the diminished surface recombination is also reflected in the corresponding characteristic transient decay times^43^ (see Figure S12d, Supporting Information), which increase from 1.1 s (BiVO_4_) to 1.5 s (**Composite 3**) to 1.7 s **(Composite 1)** up to 3.5 s for **Composite 2**. These data demonstrate the importance of surface modification by CoPOM for reducing the surface recombination kinetics and highlight the improved surface catalysis as one of the key beneficial effects of the “double-use” strategy on the overall photoanode performance.

The performance of all three photoanodes was also examined by repeated linear sweep voltammetry (LSV) under chopped illumination in an aqueous borate electrolyte at pH 9.0 (Figure S13, Supporting Information). For the modified **Composite 1** and **Composite 2** electrodes, we noticed slightly increasing photocurrents between the first and second LSV scan. We suggest that this might be due to the oxidation of the Co from Co^2+^ to Co^3+^ in the surface layer, accompanied by partial conversion of CoPOM into cobalt oxide.^26^ This was verified by performing the identical experiment with the individual Co-, W- or P-doped BiVO_4_ (Figure S14, Supporting Information), where a similar initial photocurrent increase was only observed for the Co-doped BiVO_4_ sample, while samples doped only with W or P did not show this initial increase of photocurrents. Notably, after the initial increase, photocurrents of the Co-doped BiVO_4_ then dropped (Figure S14a, Supporting Information), suggesting that this sample is not stable under the operational conditions during repeated LSV measurements. Note that in the presence of both dopants, Co and W (Figure S14e,g, Supporting Information), we observe the characteristic behavior observed for the CoPOM-doped sample, i.e. initial increase of the photocurrent between the LSV runs 1 and 2, and stable photocurrents afterward in the repeated LSV measurements. These data again corroborate our hypothesis of a combined beneficial synergistic effect of doping with both Co and W on the BiVO_4_ photoanode performance.

The wavelength-resolved IPCE was measured at *E* = 0.80 V vs. RHE both under irradiation from the back-side (BS, substrate side) and the front-side (FS, electrolyte side) (Figure 3c). The photoconversion efficiencies show a photoconversion onset at ca. 530 nm, which is in line with the optical absorption edge of all samples (Figure S8 in the Supporting Information). Under the BS illumination, which is typically beneficial for mesoporous photoanodes such as ours since the performance-limiting process is the transport of electrons through the porous network into the underlying FTO substrate, the IPCE values of both the doped and surface-modified **Composite 2** photoanode are the highest in the whole wavelength range, as shown in Figure 3c. Notably, at the doped **Composite 1** photoanode the IPCE values under the FS illumination are nearly the same as under the BS illumination (Figure 3c). This implies that the CoPOM-doping has a notable enhancing effect on the electron transport properties of BiVO_4_, and the electron transport no longer limits the photocurrents, even if the electrons have to be transported across longer pathways to the FTO support under the FS illumination.^59^ This positive effect of the CoPOM doping is further apparent from the fact that at **Composite 3** the IPCE values under the FS illumination are still slightly lower compared to the BS illumination (Figure S15a, Supporting Information). On the other hand, in the case of the **Composite 2**, the expectedly higher IPCE values under the BS illumination as compared to the FS illumination are observed, which is most probably due to a more efficient hole extraction by the cocatalyst, rendering the electron transport through the electrode limiting again.^26^

Solid-state PL spectra recorded under the 400 nm excitation wavelength (Figure S16, Supporting Information) exhibit broad bands at ca. 480–510 nm (2.4–2.6 eV) that can be attributed to band-to-band recombination, typical for monoclinic BiVO_4_ (bandgap of ca. 2.4 eV). The relative intensities for all samples are similar and do not show any definite trend, whereby the lowest intensity for **Composite 2** might suggest that the surface modification by CoPOM might have a positive effect on charge separation, as is apparent from slightly diminished radiative recombination.

To gain further insights into the factors limiting the performance of our photoanodes, the charge separation efficiency (η_sep_) and the hole transfer efficiency (η_tr_) were calculated by employing the methodology developed by Dotan et al.^40^ and Hamann et al.^41^ (see Experimental Section). According to the electronic absorption properties shown in Figure S8, Supporting Information, the *J*_max_ was calculated as 8.12 mA cm^–2^ (BiVO_4_), 7.96 mA cm^–2^ (**Composite 1**), 8.11 mA cm^–2^ (**Composite 3**) and 8.09 mA cm^–2^ (**Composite 2**). In the presence of Na_2_SO_3_ as an effective hole scavenger, the photocurrent densities reach 3.22 mA cm^–2^ (BiVO_4_), 6.32 mA cm^–2^ (**Composite 1**), 6.10 mA cm^–2^ (**Composite 3**), and 6.55 mA cm^–2^ (**Composite 2**) at 1.23 V vs. RHE, as shown in Figure S17, Supporting Information. As a result (Figure 3d), the η_sep_ for **Composite 1** and **Composite 2** photoanodes show much higher values than that for pristine BiVO_4_ in the potential ranging from +0.35 to 1.23 V vs. RHE. The η_sep_ of **Composite 1** photoanode reaches 76.5% at 1.23 V vs. RHE, which is two times higher compared to the 38.3% of the nonmodified photoanode. This indicates that CoPOM-doping effectively improves the conductivity and facilitates charge transport through the photoanode. The increase in conductivity upon CoPOM doping is also further corroborated by electrochemical impedance spectroscopy (EIS) results (for details, see Figure S19 and Table S4, Supporting Information). Interestingly, at very low bias potentials (<0.35 V vs. RHE) the detrimental effects of doping are apparent as the η_sep_ decreases as compared to pristine BiVO_4_. In our previous study on Mo-doped BiVO_4_,^26^we discussed in detail these detrimental effects of doping as related to the higher concentration of electron polaronic states due to the presence of the dopant.^60^ The electron trapping in these polaronic states limits the maximum achievable photovoltage and enhances the charge recombination, unless a sufficiently high bias is applied. Indeed, the negative effect of the CoPOM-doping is clearly corroborated by the reduced quasi-Fermi level of electrons after the photodoping, as apparent from reduced open-circuit photopotentials at **Composite 1** and **Composite 2** as compared to pristine BiVO_4_ (see Figure S18a, Supporting Information). Furthermore, the hole transfer efficiency η_tr_ (Figure 3e) is significantly enhanced over the whole potential range and shifted to cathodic potentials for both modified photoanodes. Specifically, the η_tr_ increased from 12.7% for BiVO_4_ to 47.8% for **Composite 1** and 59.2% for **Composite 2** photoanodes at 1.23 V vs. RHE. Notably, the beneficial effects of the CoPOM cocatalyst become particularly significant at very low bias potentials (<0.45 V vs. RHE), where it can partially compensate the detrimental effects of the electron polaronic states from doping. Interestingly, the η_sep_ for **Composite 1** and **Composite 3** photoanodes show a similar value over the whole potential range, while the value of η_tr_ is higher for **Composite 1** than that of **Composite 3** (Figure S15b,c, Supporting Information). This hints that hole transfer efficiency η_tr_ plays an important role in PEC performance and is in line with the difference of ratio of W/Co on the surface (see Table S1, Supporting Information). Taken together, all these findings indicate that the “double-use” of CoPOM as both a dopant and a surface cocatalyst can effectively promote the charge separation and hole transfer carrier dynamics at BiVO_4_ photoanodes, respectively, in line with the established knowledge on the effects of doping and cocatalysts in the literature.^25,38^

**Figure 4:**
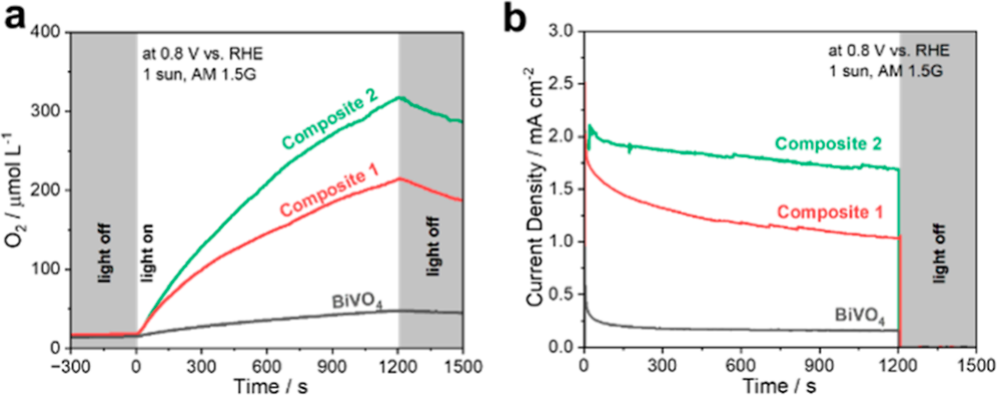
(a) Oxygen evolution and (b) corresponding photocurrent transients recorded under AM 1.5G at 0.80 V vs. RHE in a borate buffer for BiVO_4_ and **Composite 1** and **Composite 2** photoanodes.

To confirm the actual oxygen evolution performance of the photoanodes, we performed photoelectrocatalytic OER measurements (Figure 4a) in an aqueous borate solution (pH 9.0) under AM 1.5G (1 sun) illumination at *E* = 0.80 V vs. RHE. Under these conditions, we observed O_2_ evolution rates of 104.1 ± 18.5 μmol L^–1^ h^–1^ (BiVO_4_), 504.7 ± 26.7 μmol L^–1^ h^–1^ (**Composite 1**), and 856.9 ± 33.3 μmol L^–1^ h^–1^ (**Composite 2**) during a 20 min illumination experiment. The corresponding faradaic efficiencies are comparable for the three systems (BiVO_4_:102.7% ± 13.2%; **Composite 1**: 97.5% ± 9.5%, **Composite 2**: 102.3% ± 12.2%), indicating the nearly quantitative oxygen evolution at all BiVO_4_ photoanodes.

**Figure 5:**
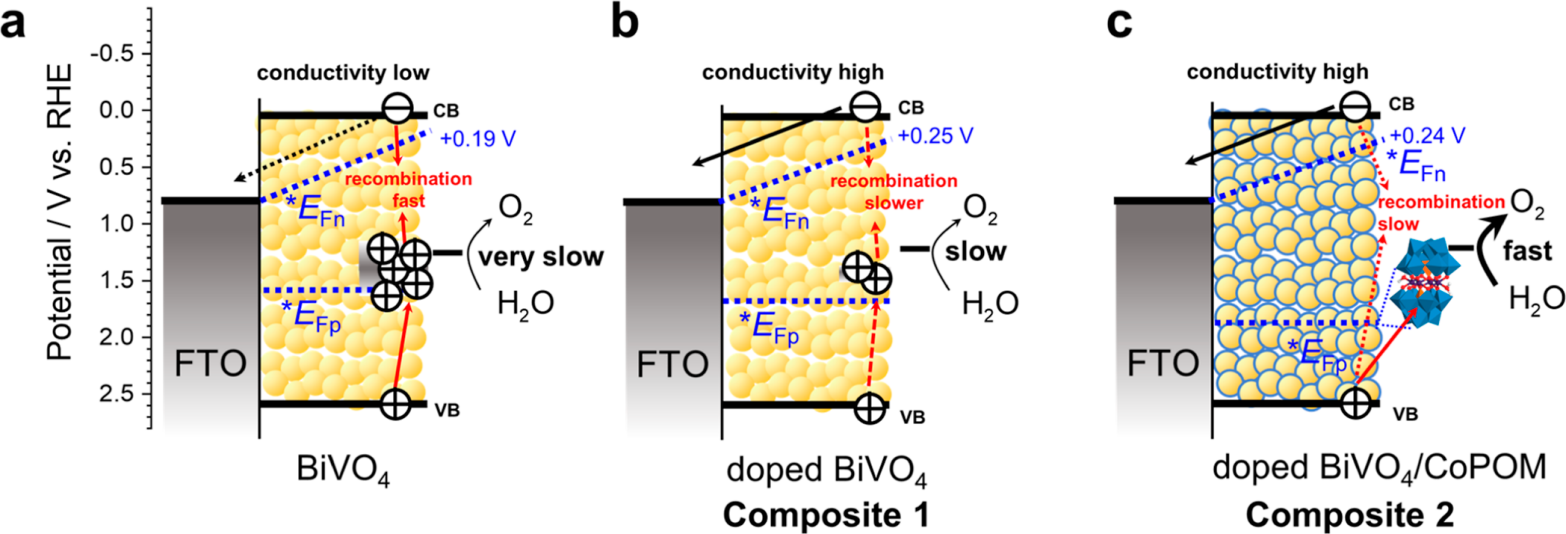
Simplified scheme summarizing the effect of the “double-use” strategy employing CoPOM on photoanodes at a moderate electrode bias of +0.80 V vs. RHE: (a) pristine BiVO_4_ suffers both from ineffective electron transport through the mesoporous film and the accumulation of holes in surface states, resulting in enhanced recombination and very low photocurrents (compare with Figure 3a); (b) doped BiVO_4_ (**Composite 1**) exhibits slower recombination due to higher conductivity and less pronounced hole accumulation; and (c) doped BiVO_4_ surface-modified with CoPOM (**Composite 2**) exhibits the highest photocurrents due to both the high conductivity and the effective hole extraction and enhanced water oxidation by the CoPOM-derived surface cocatalyst. Note that the band bending is assumed to be negligible due to the mesoporous nature of the BiVO_4_ film. CB and VB stand for the conduction band edge and valence band edge, respectively; *E_Fn_ and *E_Fp_ stand for the quasi-Fermi level of electrons and holes in the mesoporous film, respectively. The maximum values of *E_Fn_ were taken as the maximum open-circuit photopotentials from the Figure S18a in the Supporting Information.

Finally, the ABPE was also determined. As shown in Figures 3f and S15d, the Supporting Information, the maximum photoconversion efficiencies are 0.03% (at 1.04 V vs. RHE) for BiVO_4_, 0.39% (at 0.94 V vs. RHE) for **Composite 3**, 0.54% (at 0.87 V vs. RHE) for **Composite 1** and 0.79% (at 0.82 V vs. RHE) for **Composite 2**. Two points are noteworthy. First, the fact that the maximum power point is shifted to more negative bias potentials after doping and surface modification with CoPOM is highly beneficial in view of the potential construction of tandem solar-driven water-splitting systems in which photoanodes for O_2_ evolution are combined with photocathodes for H_2_ evolution. Second, the maximum ABPE is 0.79% (at 0.82 V vs. RHE) for the **Composite 2** is not only higher by a factor of ∼26 as compared to pristine BiVO_4_, but even slightly higher than the best performing Mo-doped BiVO_4_ photoanode modified with CoPOM cocatalyst (ABPE of 0.73% at 0.86 V vs. RHE) we investigated recently.^26^ This highlights the beneficial effect of the “double-use” strategy employing CoPOM on the fabrication of BiVO_4_ photoanodes with improved performance for a comprehensive comparative overview of key PEC performance factors of all photoanodes (see Table S5, Supporting Information). The key factors influencing the PEC performance of all electrodes at a moderate electrode bias of 0.80 V vs. RHE are summarized in Figure 5.

## 4 Conclusions

In conclusion, we have demonstrated a novel “double-use” strategy for the fabrication of high-performance BiVO_4_ photoanodes for solar water splitting in which a single precursor, a molecular cobalt polyoxometalate (CoPOM), is used both as a doping agent and as a cocatalyst for water oxidation. Doping BiVO_4_ by CoPOM significantly enhances the conductivity and improves the charge separation efficiency, resulting in the enhancement of the maximum ABPE by a factor of ∼18 as compared to that of pristine BiVO_4_. Further surface modification of CoPOM-doped BiVO_4_ with CoPOM as a cocatalyst enhances the hole extraction and improves the water oxidation kinetics, yielding the overall enhancement of the ABPE as high as by a factor of ∼26 with respect to pristine BiVO_4_. Interestingly, with respect to the doped BiVO_4_ photoanodes, we found systematic differences in the surface composition and PEC performance of samples doped with CoPOM compared to samples with identical elemental composition but doped with metal salts. While these differences are not fully understood yet, they point to possible advantages of using molecularly well-defined precursors, such as CoPOM, in the fabrication of high-performance photoelectrodes. Taken together, our results establish the “double-use” strategy involving CoPOMs as a remarkably effective, straightforward, and easily scalable approach for the development of high-quality photoanodes for solar water splitting and highlight the future potential of utilizing well-designed POMs as precursors for the synthesis of energy materials.

## Supporting Information Available

Additional analytical, characterization, and electrochemical data (PDF)

## Notes

All data sets shown in this study are available from zenodo.org under the link 10.5281/zenodo.13882299.
