# Correction to “Double-Use Strategy for Improving the Photoelectrochemical Performance of BiVO_4_ Photoanodes Using a Cobalt-Functionalized Polyoxotungstate”

**DOI:** 10.1021/acsami.5c05735

**Published:** 2025-04-03

**Authors:** Fan Feng, Dariusz Mitoraj, Ekemena Oseghe, Carsten Streb, Radim Beranek

In our original paper, in the Introduction, in the sentence “As a result, the reported photocurrent densities at nonmodified BiVO_4_ are typically much lower than the maximum theoretical value of 6.4–8.9 mA cm^–2^ under AM 1.5G illumination, corresponding to maximum solar-to-H_2_ conversion efficiencies of 8–11%.”, the values relating to the maximum theoretically achievable photocurrents and solar-to-hydrogen (STH) efficiencies of BiVO_4_ photoanodes were incorrectly calculated.

The sentence should read “As a result, the reported photocurrent densities at nonmodified BiVO_4_ are typically much lower than the maximum theoretical value of 5.1–7.5 mA cm^–2^ under AM 1.5G illumination, corresponding to maximum solar-to-H_2_ conversion efficiencies of 6.2–9.3%.”

These errors do not affect the results or conclusions of this article.

