# Supplementary material for: “Double-Use” Strategy for Improving the Photoelectrochemical Performance of BiVO4 Photoanodes Using a Cobalt-Functionalized Polyoxotungstate
Source: ACS Appl Mater Interfaces. 2024 Dec 30;17(2):3665–75. doi: 10.1021/acsami.4c21125 (PMC11744501; doi:10.1021/acsami.4c21125)
Supplement: Supplementary file 1 — am4c21125_si_001.pdf [file am4c21125_si_001.pdf]

SUPPORTING INFORMATION

"Double-Use" Strategy for Improving the Photoelectrochemical Performance of $BiVO_4$ Photoanodes using a Cobalt-Functionalized Polyoxotungstate

Fan Feng,[†] Dariusz Mitoraj,[‡] Ekemena Oseghe,[†] Carsten Streb,[*,†] Radim Beranek[*,‡]

[†] Department of Chemistry, Johannes Gutenberg University Mainz, Duesbergweg 10-14, 55128 Mainz, Germany
[‡] Institute of Electrochemistry, Ulm University, Albert-Einstein-Allee 47, 89081 Ulm, Germany
* Corresponding authors: radim.beranek@uni-ulm.de, carsten.streb@uni-mainz.de

Analytical section:

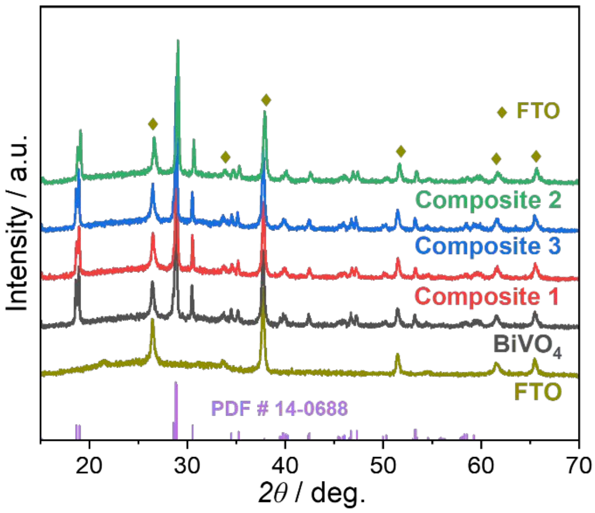

Figure S1: XRD patterns of $BiVO_4$, **Composite 1**, **Composite 3**, **Composite 2** and FTO substrate.

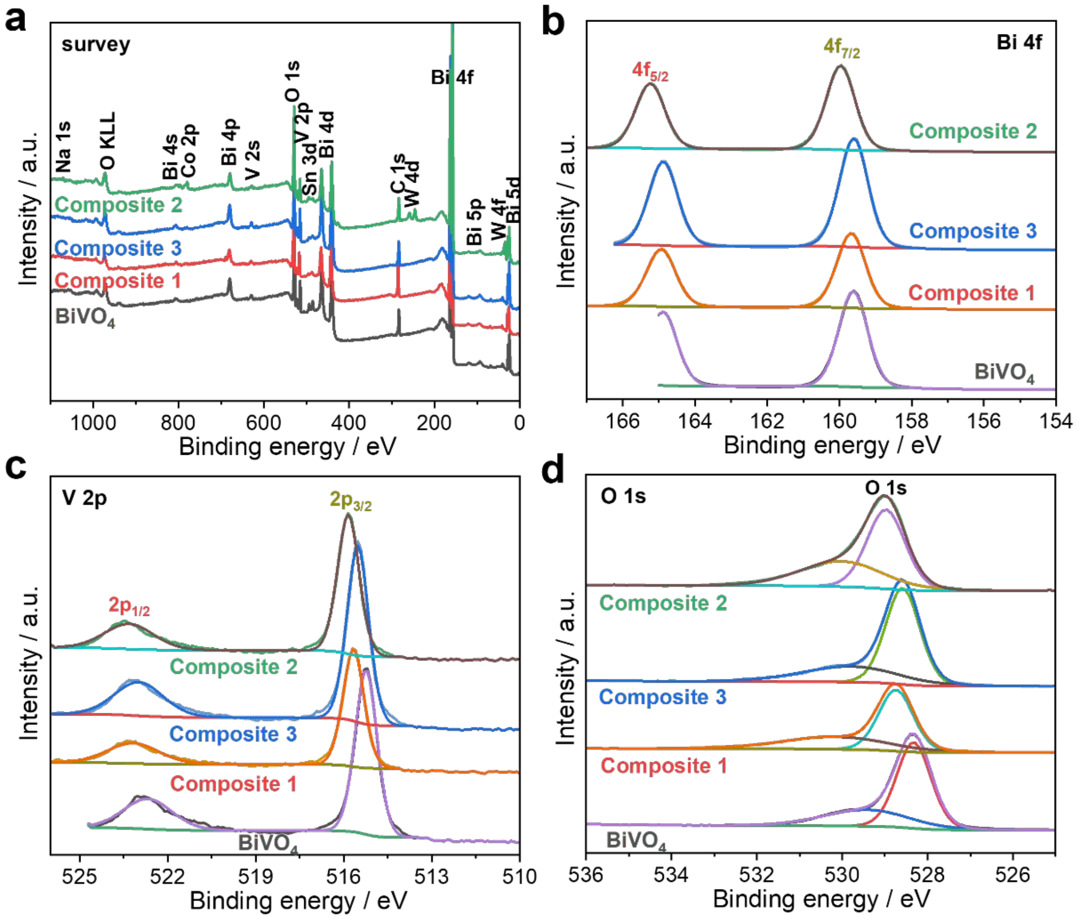

Figure S2: XPS spectroscopic analysis of all photoanodes reported: (a) survey spectra, (b) deconvoluted Bi 4f region, (c) deconvoluted V 2p region, (d) deconvoluted O 1s region.

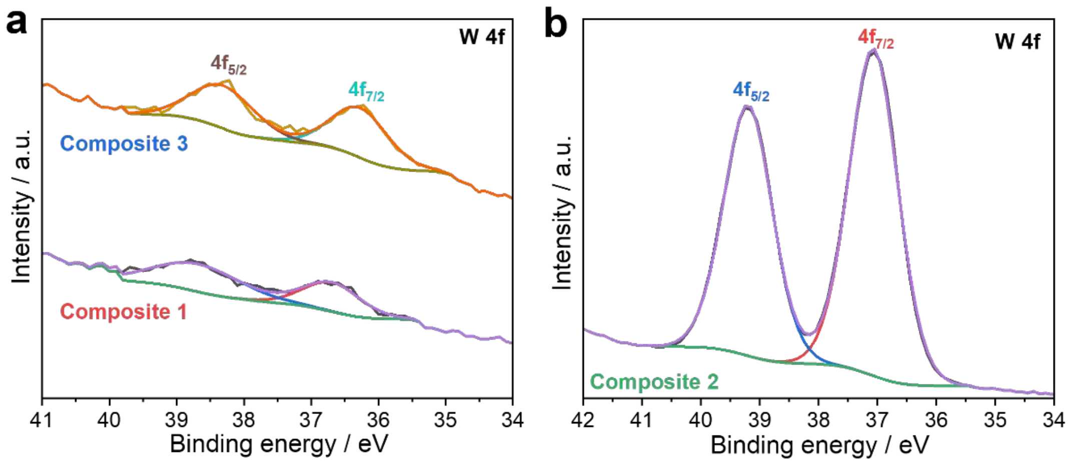

Figure S3: W 4f XP spectra for (a) **Composite 1** and **Composite 3**, (b) **Composite 2** photoanodes.

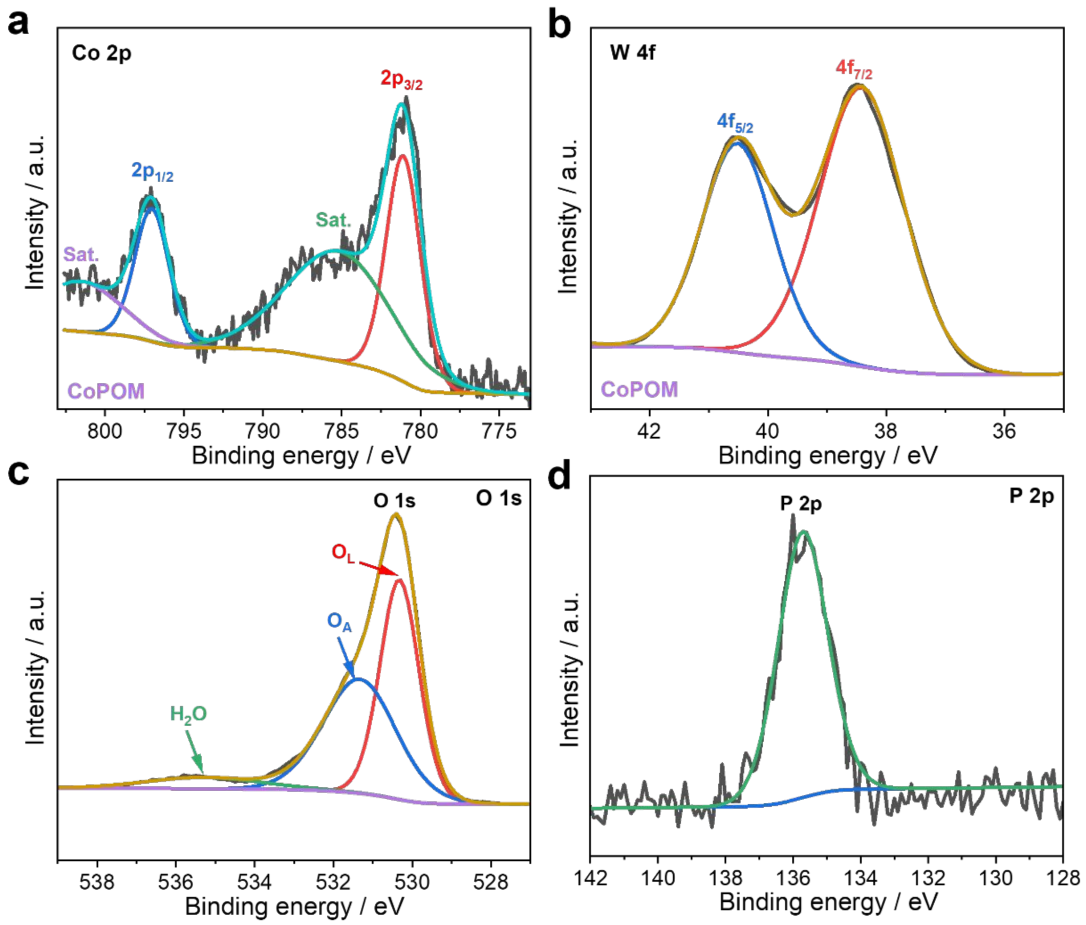

Figure S4: XP spectra of CoPOM powder (a) Co 2p, (b) W 4f, (c) O 1s and (d) P 2p.

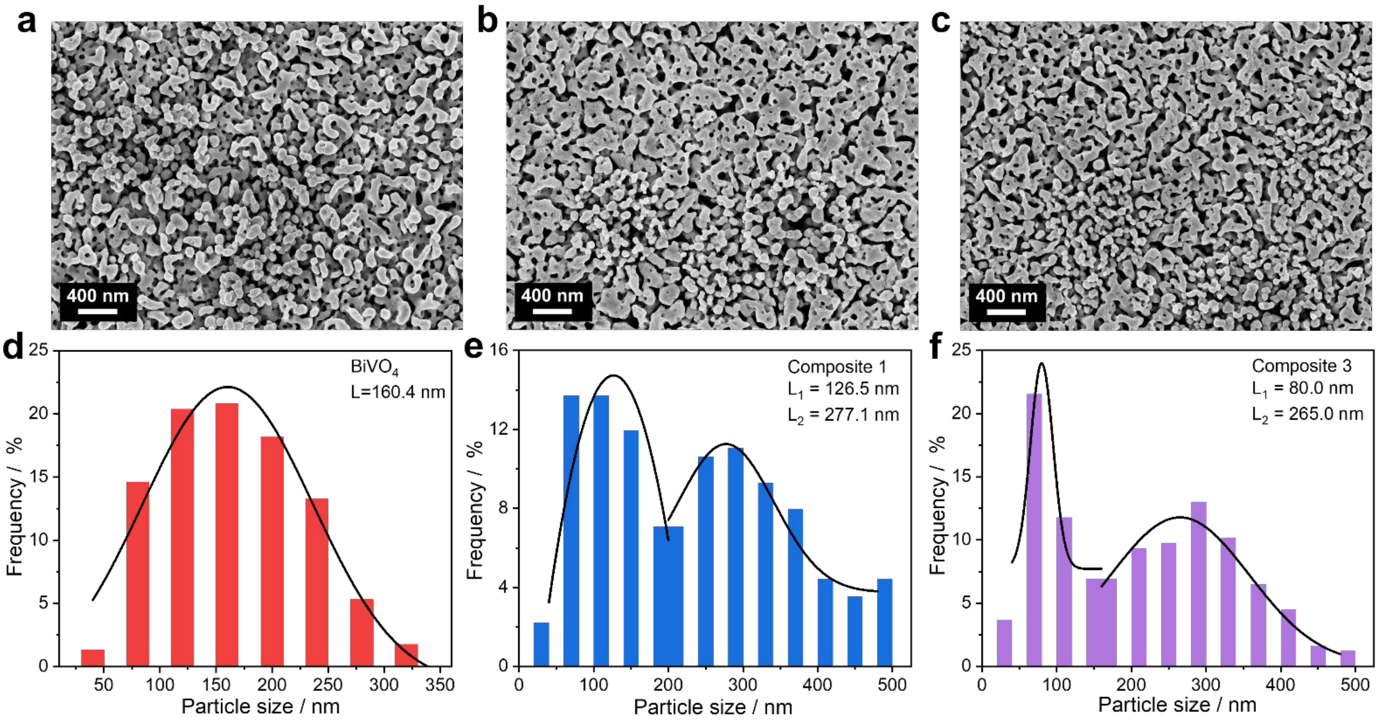

Figure S5: SEM images of (a) $BiVO_4$, (b) **Composite 1** and (c) **Composite 3**. Diagrams of particle size distribution statistics of (d) $BiVO_4$, (e) **Composite 1** and (f) **Composite 3**.

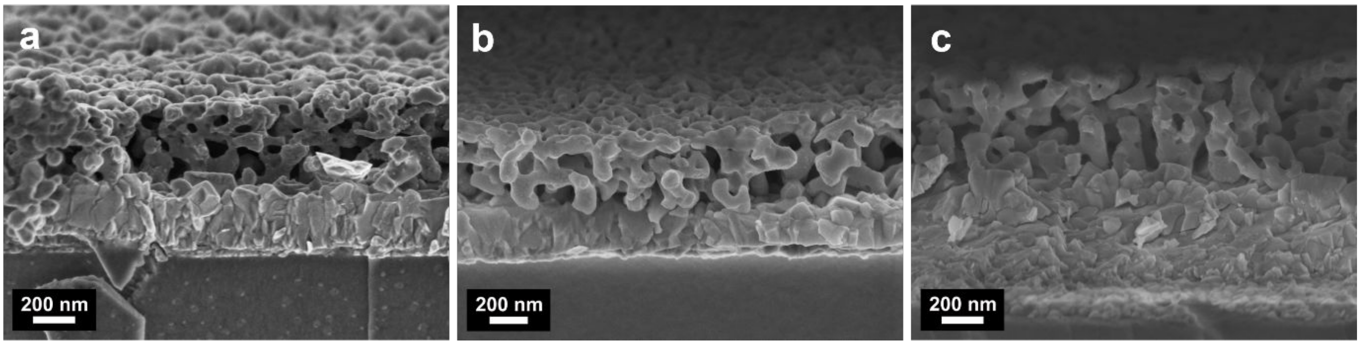

Figure S6: SEM cross-section images of (a) $BiVO_4$, (b) **Composite 1** and (c) **Composite 2**. The x-section images were taken 75° pre-tilt for all photoanodes.

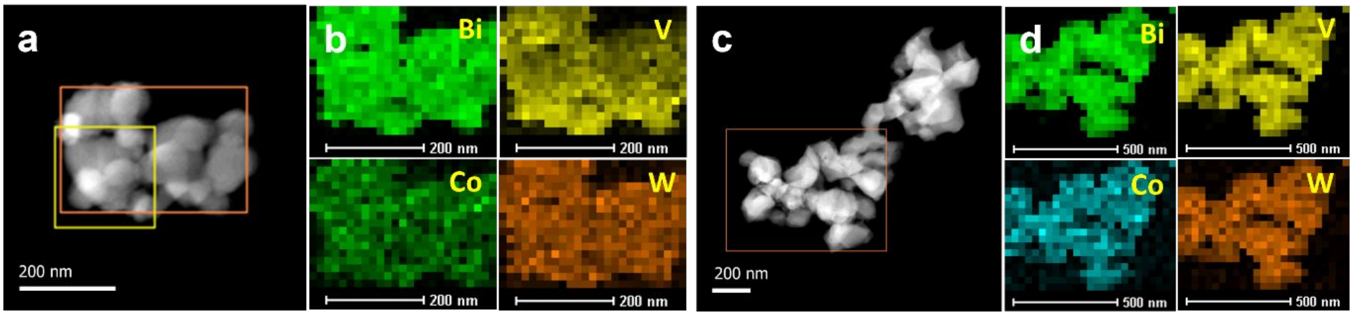

Figure S7: STEM elements mapping of (a, b) **Composite 1** and (c, d) **Composite 2** photoanodes.

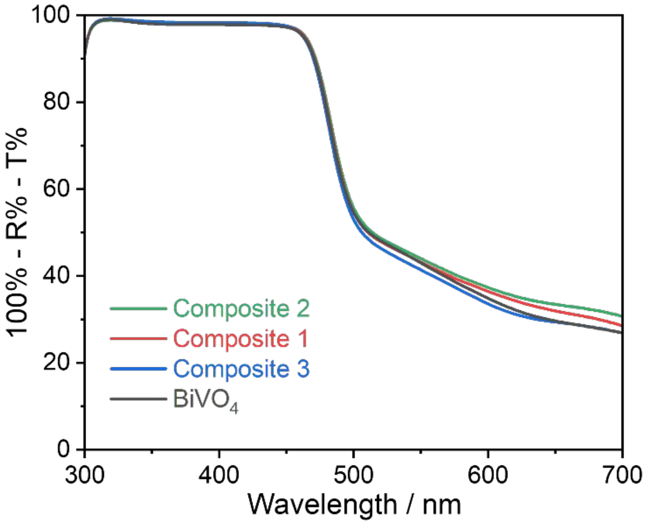

Figure S8: UV-Vis electronic absorption spectra of the photoanodes. $Absorptance$ (%) = 100% − $Reflectance$ (%) − $Transmittance$ (%).

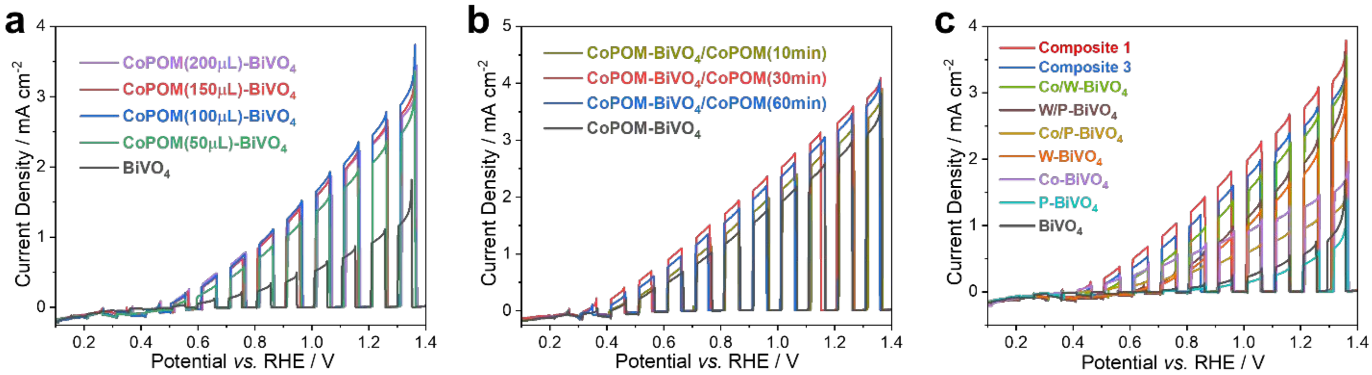

Figure S9: J-V curves of (a) different amount for CoPOM doping on $BiVO_4$ photoanodes; (b) different amount for CoPOM on CoPOM-$BiVO_4$ phtoanodes; (c) different element-doping with the same molar amount with CoPOM doping samples in sodium borate buffer electrolyte under AM 1.5G (1 sun).

The photocurrent density of P-$BiVO_4$ and Co/P-$BiVO_4$ are slightly decreased compared to the P-doped-free samples, while the photocurrent density of W/P-$BiVO_4$ shows a slight increase trend compared with W-$BiVO_4$ photoanodes, suggestion is that W-doped can be able to a stable factor for doped $BiVO_4$-based samples.

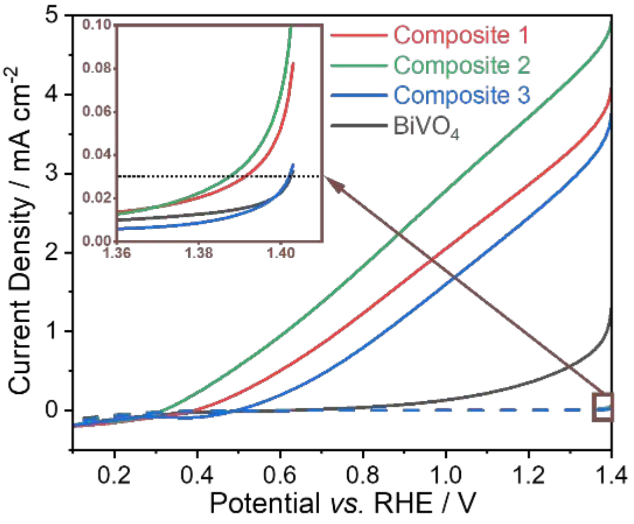

Figure S10: LSV curves of $BiVO_4$, **Composite 1, Composite 3** and **Composite 2** in borate buffer electrolyte pH 9.0 under AM 1.5G (1 sun) illumination. Dotted curves represent measurements carried out in the dark, and the inset shows an enlarged view of curves measured in the dark. The lowest overpotential of 158 mV (@ j = 0.03 mA cm^{-2}) is observed for **Composite 2** compared to that of **Composite 1** (161 mV), **Composite 3** (172 mV) and pure $BiVO_4$ (173 mV).

Table S1: concentration (in at.%) of the detected elements in XPS

Samples	Bi	V	O	C	Co	W	P	Na	Ratio (W/Co)
$BiVO_4$	14.35	7.14	49.14	29.13	-	-	-	0.24	-
Composite 1	10.00	5.44	36.43	47.90	0.07	0.16	0.00	0.01	2.29
Composite 3	13.76	7.33	48.90	29.52	0.24	0.26	0.00	0.00	1.08
Composite 2	11.23	5.86	53.75	25.99	1.28	1.42	0.00	0.48	1.11

Table S2: concentration (in at.%) of the detected elements in XPS for **Composite 1** before PEC and after PEC.

Samples	Bi	V	O	C	Co	W	P	Na	Ratio (W/Co)
Before	10.00	5.44	36.43	47.90	0.07	0.16	0.00	0.01	2.29
Normalized (Before)	19.19	10.44	69.92	-	0.13	0.30	0.00	0.02	2.31
After	13.76	5.98	46.17	32.82	0.68	0.40	0.00	0.18	0.59
Normalized (After)	20.48	8.90	68.73	-	1.02	0.60	0.00	0.27	0.59

Table S3: concentration (in at.%) of the detected elements in XPS for **Composite 2** before PEC and after PEC.

Samples	Bi	V	O	C	Co	W	P	Na	Ratio (W/Co)
Before	11.23	5.86	53.75	25.99	1.28	1.42	0.00	0.48	1.11
Normalized (Before)	15.17	7.92	72.62	-	1.73	1.92	0.00	0.65	1.11
After	10.46	5.72	46.50	34.65	1.53	1.10	0.00	0.04	0.72
Normalized (After)	16.01	8.75	71.16	-	2.34	1.68	0.00	0.06	0.72

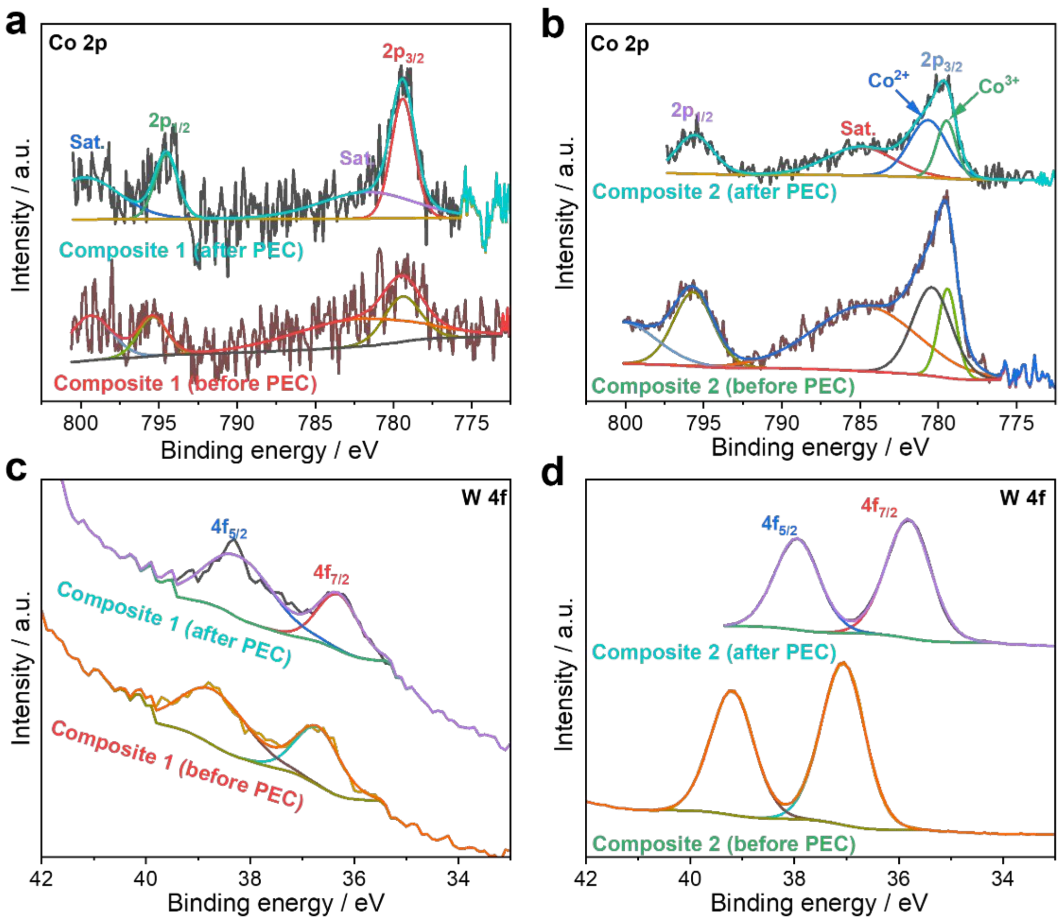

Figure S11: Co 2p XP spectra for (a) **Composite 1**, (b) **Composite 2** and W 4f XP spektra for (c) **Composite 1**, (d) **Composite 2** before and after PEC stability experiments (4 h).

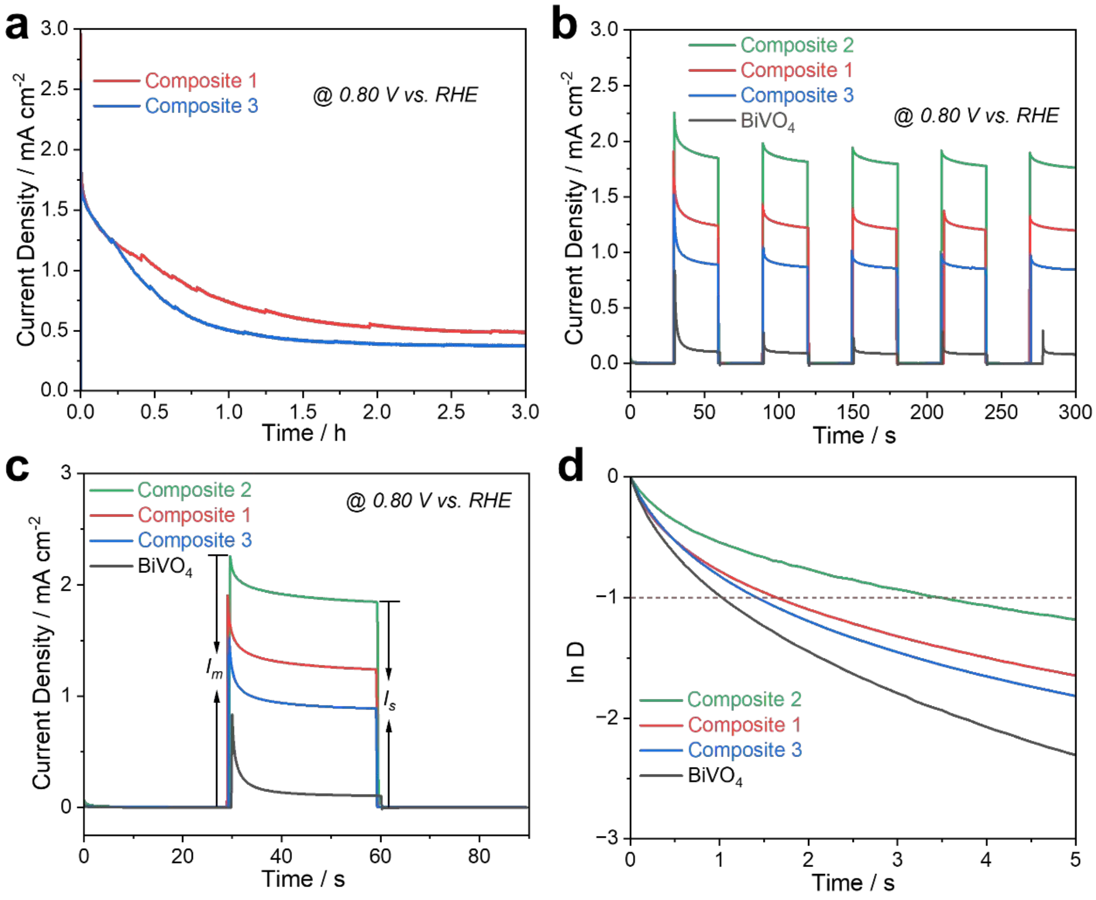

Figure S12: (a) Chronoamperometry curves under AM 1.5G illumination at 0.80 V *vs*. RHE for **Composite 1** and **Composite 3**. (b, c) Photocurrent transients under AM 1.5G (1 sun) illumination at +0.80 V *vs*. RHE, and (d) transient decay times (taken as time at ln D = –1) of $BiVO_4$, **Composite 1**, **Composite 2** and **Composite 3**.

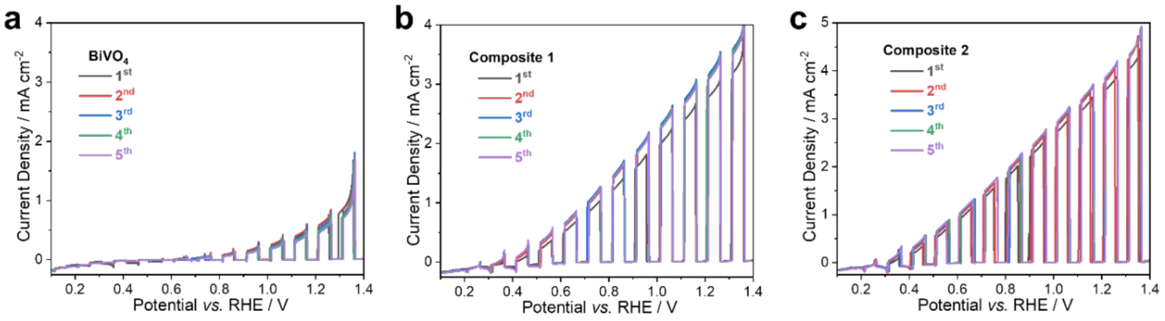

Figure S13: Photocurrent stability test recorded under AM 1.5G (1 sun) illumination at anodic sweep of 10 mV s^{-1} for (a) $BiVO_4$, (b) **Composite 1**, and (c) **Composite 2** samples.

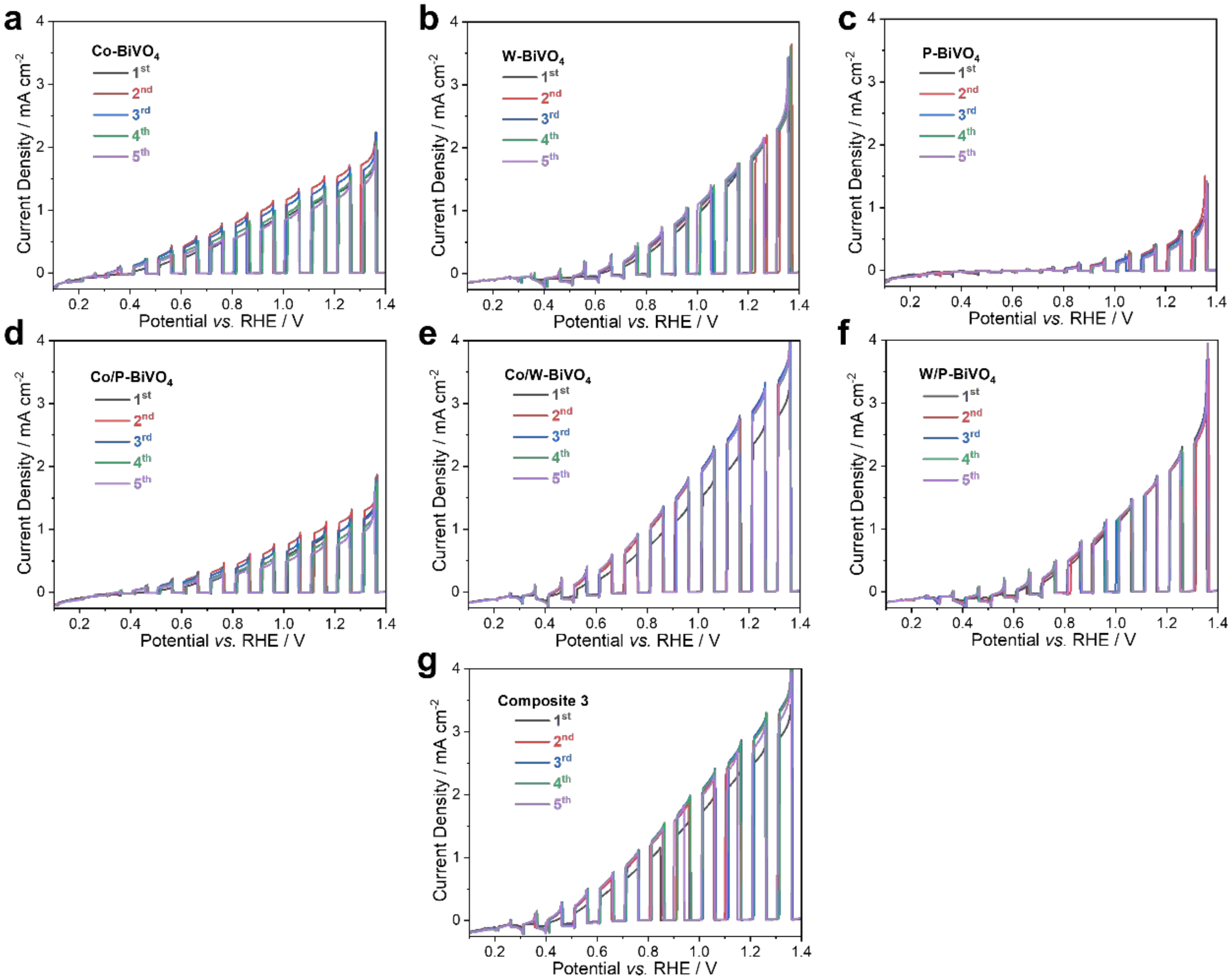

Figure S14: Photocurrent stability test recorded under AM 1.5G illumination at anodic sweep of 10 mV s^{-1} for (a) Co-$BiVO_4$, (b) W-$BiVO_4$, (c) P-$BiVO_4$, (d) Co/P-$BiVO_4$, (e) Co/W-$BiVO_4$, (f) W/P-$BiVO_4$ and (g) **Composite 3** samples.

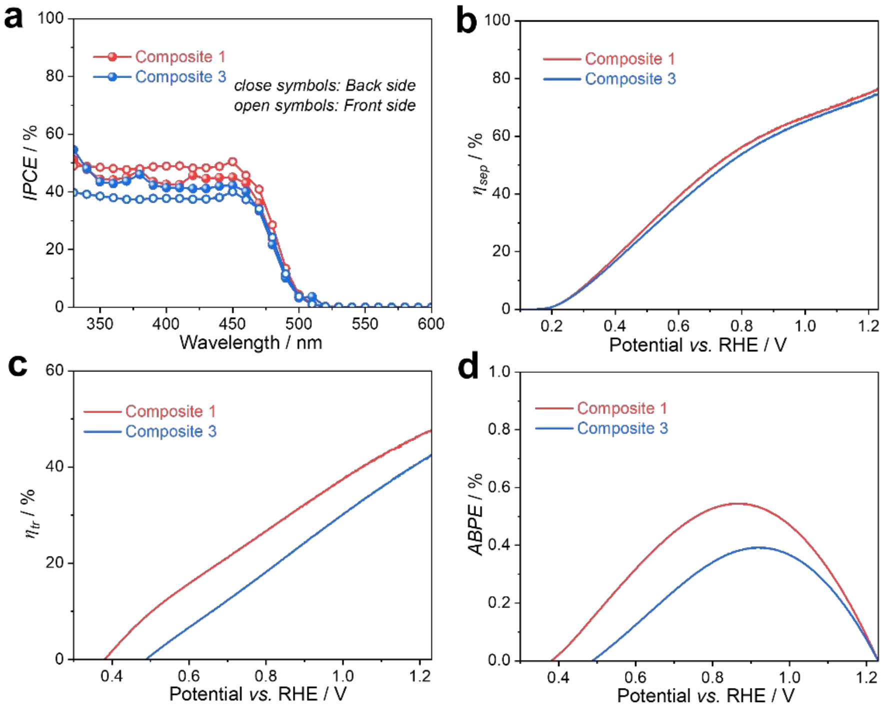

Figure S15: (a) IPCE spectra under intermittent monochromatic irradiation; (b) Charge separation efficiency (η_{sep}), (c) hole transfer efficiency (η_{tr}) and (d) applied bias photoconversion efficiency (ABPE) plots for **Composite 1** and **Composite 3** photoanodes.

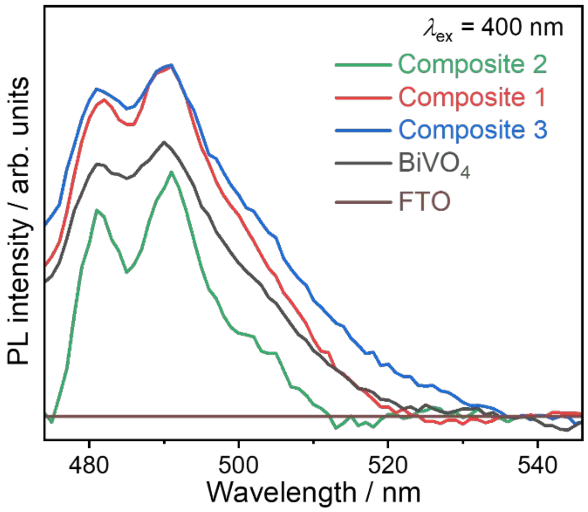

Figure S16: PL spectra (after abstraction of the baseline due to the FTO response) of FTO, $BiVO_4$, **Composite 1**, **Composite 2** and **Composite 3** photoanodes recorded at the excitation wavelength of 400 nm.

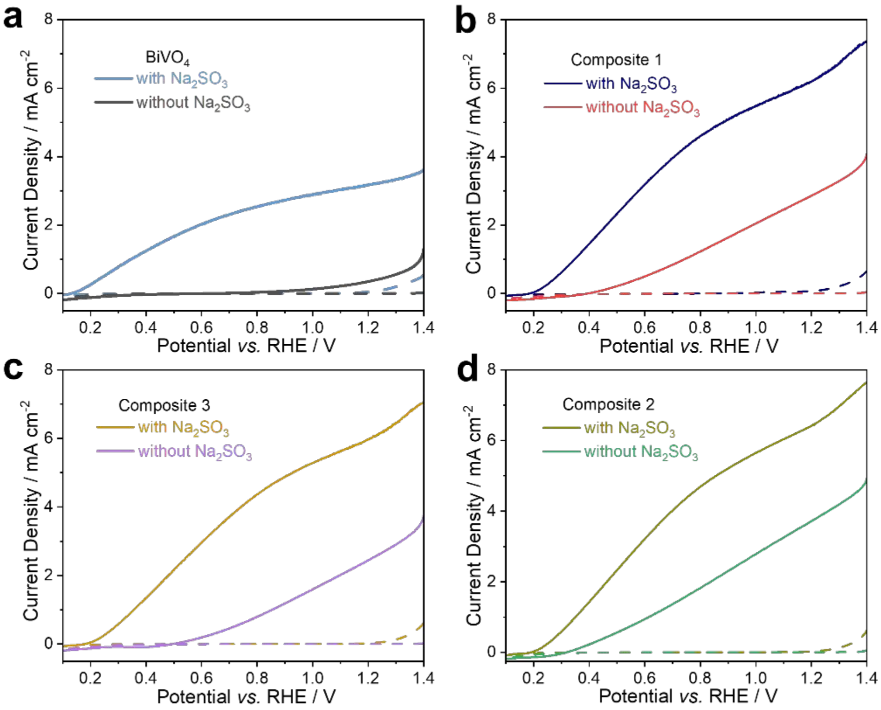

Figure S17: J-V curves of (a) $BiVO_4$, (b) **Composite 1,** (c) **Composite 3** and (d) **Composite 2** in borate buffer electrolyte pH 9.0 with and without 0.1 M Na_2SO_3 under AM 1.5G (1 sun) illumination. Dotted curves represent measurements in the dark.

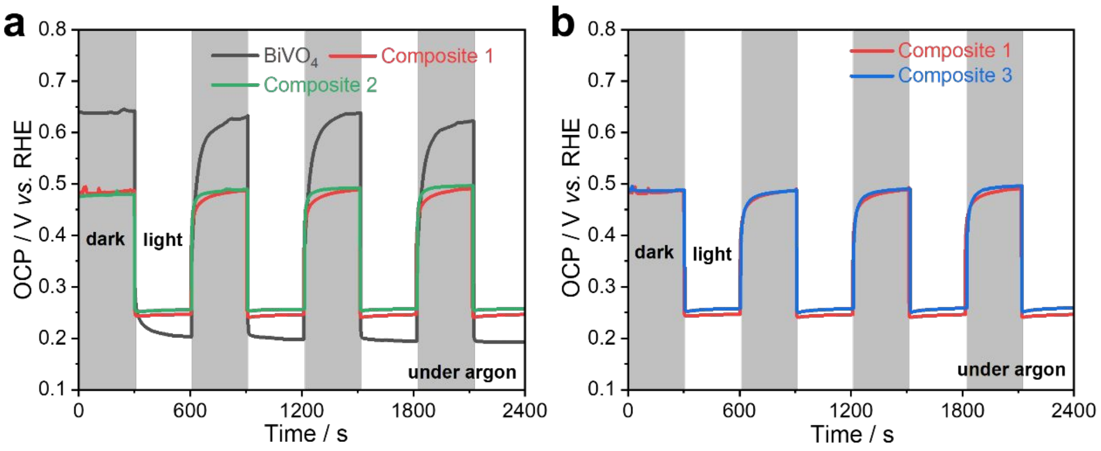

Figure S18: Open-circuit potential transients recorded under AM 1.5G in 0.5 M borate buffer under argon atmosphere for (a) $BiVO_4$, **Composite 1** and **Composite 2**, and (b) **Composite 1** and **Composite 3**. The electrodes were irradiated from the back-side.

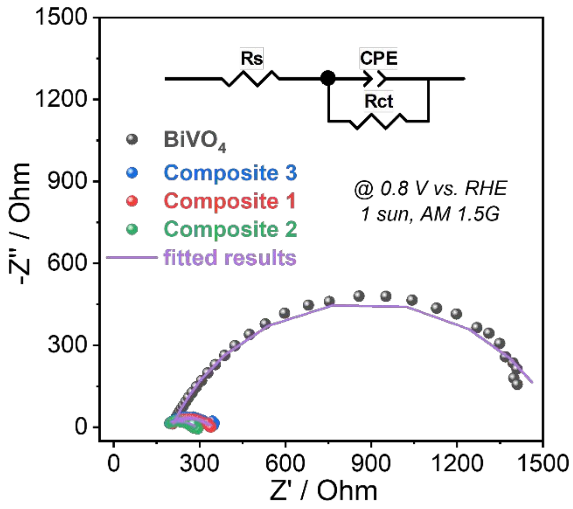

Figure S19: EIS curves (inset: equivalent circuit used for fitting) of $BiVO_4$, **Composite 1, Composite 2** and **Composite 3** in 0.5 M borate buffer electrolyte pH 9.0 under AM 1.5G 1 sun illumination. Solid curves represent the fitted results.

Table S4: The fitted results of EIS data using the equivalent circuit in Fig. S19.

Samples	R_s (Ω)	R_{ct} (Ω)
**Composite 2**	158.5 ± 16.1	130.4 ± 19.1
**Composite 1**	185.8 ± 2.8	153.7 ± 4.4
**Composite 3**	174.3 ±4.4	174.4 ± 6.5
$BiVO_4$	203.5 ± 1.9	1353.0 ± 18.4

For simplicity, we used a Randles-type equivalent circuit model, where R_s represents the uncompensated series resistance, R_{ct} in our case represents the combined charge transport and interfacial charge transfer resistance and CPE is the constant phase element for the electrode/electrolyte interface. The fitted values of R_s and R_{ct} are shown in Table S4. The similar Rs values of four films indicate that the effect of series resistance is negligible, while the values of R_{ct} of **Composite 1** (~154 Ω), **Composite 3** (~174 Ω) and **Composite 2** (~130 Ω) decrease significantly as compared to pristine $BiVO_4$ (~1353 Ω), indicating that 'Double-Use' of CoPOM greatly enhances both charge transport (i.e., conductivity) and the charge transfer, resulting in enhanced photocurrent density. Additionally, the value of R_{ct} of **Composite 1** is slightly smaller than that of **Composite 3**, which is also a factor to explain the slightly enhanced photocurrent density.

Table S5: The comparison of photoelectrochemical performance.

Samples	**Composite 2**	**Composite 1**	**Composite 3**	$BiVO_4$
Photocurrent density @ 1.23 V *vs.* RHE	3.67 mA cm^{-2}	2.86 mA cm^{-2}	2.57 mA cm^{-2}	0.62 mA cm^{-2}
Onset potential	~ 0.32 V *vs.* RHE	~ 0.39 V *vs.* RHE	~ 0.50 V *vs.* RHE	~ 0.61 V *vs.* RHE
η_{sep} @ 1.23 V vs. RHE	79.3%	76.5%	74.7%	38.3%
η_{tr} @ 1.23 V vs. RHE	59.2%	47.8%	42.5%	12.7%
ABPE (maximum value)	0.79% @ 0.82 V *vs.* RHE	0.54% @ 0.87 V *vs.* RHE	0.39% @ 0.94 V *vs.* RHE	0.03% @ 1.04 V *vs.* RHE
IPCE @ 450 nm	BS: 51.7% FS: 46.9%	BS: 45.1% FS: 50.5%	BS: 42.3% FS: 40.0%	BS: 5.9% FS: 2.1%
